# Transcriptomic analysis reveals differential gene expression, alternative splicing, and novel exons during mouse trophoblast stem cell differentiation

**DOI:** 10.1186/s13287-020-01848-8

**Published:** 2020-08-06

**Authors:** Rahim Ullah, Ambreen Naz, Hafiza Sara Akram, Zakir Ullah, Muhammad Tariq, Aziz Mithani, Amir Faisal

**Affiliations:** 1 Department of Biology, Syed Babar Ali School of Science and Engineering, Lahore University of Management Sciences, Lahore, Pakistan; 2 Virginia Commonwealth University, Richmond, USA

**Keywords:** Trophoblast stem cells, Trophoblast giant cells, Placental development, RNA-seq, Stem cell differentiation, Alternative splicing, Differential expression

## Abstract

**Background:**

Differentiation of mouse trophoblast stem cells (TSCs) to trophoblast giant cells (TGCs) has been widely used as a model system to study placental development and function. While several differentially expressed genes, including regulators of TSC differentiation, have been identified, a comprehensive analysis of the global expression of genes and splice variants in the two cell types has not been reported.

**Results:**

Here, we report ~ 7800 differentially expressed genes in TGCs compared to TSCs which include regulators of the cell cycle, apoptosis, cytoskeleton, cell mobility, embryo implantation, metabolism, and various signaling pathways. We show that several mitotic proteins, including Aurora A kinase, were downregulated in TGCs and that the activity of Aurora A kinase is required for the maintenance of TSCs. We also identify hitherto undiscovered, cell-type specific alternative splicing events in 31 genes in the two cell types. Finally, we also report 19 novel exons in 12 genes which are expressed in both TSCs and TGCs.

**Conclusions:**

Overall, our results uncover several potential regulators of TSC differentiation and TGC function, thereby providing a valuable resource for developmental and molecular biologists interested in the study of stem cell differentiation and embryonic development.

## Background

Cellular differentiation is one of the key processes in mammalian development through which complex cell types and tissues form during embryogenesis. At the blastocyst stage of embryonic development, a single layer of polarized epithelial cells, called trophectoderm, is differentiated and segregated from the inner cell mass [1]. Trophectoderm exclusively gives rise to the placenta, whereas the inner cell mass produces the rest of the embryo [2, 3].

Mouse trophoblast stem cells (TSCs) are derived from the polar trophectoderm of the blastocyst [1] and have been widely used to study placental development [4–6]. TSCs differentiate into large, polyploid trophoblast giant cells (TGCs), which express specific sets of marker genes not found in TSCs [7, 8]. The differentiation of TSCs into TGCs is regulated by a number of factors including cytokines, hormones, protein kinases, transcription factors, proteases, and membrane proteins [9–16]. Removal of fibroblast growth factor 4 (FGF4) from TSC culture media, for example, causes them to differentiate into polyploid TGCs. FGF4 deprivation of TSCs results in downregulation of Cdk1 and Chk1, two kinases critical for cell cycle regulation. This leads to the terminal differentiation of TSCs to TGCs and paves the way for the acquisition of polyploidy without triggering apoptosis. The process of TSC differentiation is facilitated by the upregulation of CDK specific inhibitors, p57 and p21. While p57 localizes in the nucleus and inhibits TGCs from undergoing mitosis by inhibiting CDK1, p21 is localized to the cytoplasm and prevents TGCs from undergoing apoptosis [15–17]. Suppression of p21 and p57 expression by Chk1 prevents the premature differentiation of TSCs into TGCs. Treatment of TSCs with CDK1 inhibitor (RO3306), retinoic acid, or estrogen diethylstilbestrol (DES) also promote their differentiation into TGCs [15, 18, 19]. Similarly, hypoxic stress can induce irreversible differentiation of mouse TSCs, even in the presence of FGF4 in the culture medium [5]. Although several factors have been identified which can trigger differentiation of TSCs into TGCs, a broader understanding of the underlying molecular mechanisms leading to TSC differentiation remains elusive.

Human choriocarcinoma cell line BeWo provides another in vitro model of cytotrophoblast differentiation. In response to certain stimuli (e.g., elevated cAMP levels), BeWo cells fuse to form a syncytium, a phenomenon that mimics the fusion of cytotrophoblast cells to form syncytiotrophoblast. Interestingly, while CDK1 inhibition triggers differentiation of TSCs to TGCs, its inhibition in BeWo cells triggers the process of cell fusion pointing to both similarities and differences in the two systems [20]. While there is no comprehensive study that deciphers the molecular basis of mouse TSC differentiation, the fusion of BeWo cells has been studied in detail focusing on transcriptomic and epigenomic analysis using RNA seq, genome-wide DNA methylation, and ChIP seq data, respectively. The RNA-seq analysis during fusion of BeWo cells revealed alterations in the expression of ∼ 3000 genes, enriched for syncytialization, cell differentiation, morphogenesis, blood vessel and placental labyrinth development, and steroid hormone response. Similarly, genome-wide DNA methylation analysis using RRBS (reduced representation bisulfite sequencing) determined altered methylation of CpGs associated with cell lineage commitment and differentiation. ChIP-seq analysis revealed the association of syncytialization with transcriptionally active marks among genes that are upregulated [21]. Considering the importance of trophoblast differentiation in mammalian development, it is imperative to perform similar studies in the mouse TSC/TGC system which can result in the identification of key regulators of the differentiation process in a physiological context that may be over-shadowed in the BeWo cancer cell line.

In this study, we examined the gene expression profile of undifferentiated TSCs and differentiated TGCs through next-generation sequencing to determine differentially expressed genes and delineate the mechanism of differentiation. Our results have revealed the differential expression of nearly 7800 genes in TSCs/TGCs, including some which were previously known and have been used as markers of differentiation. We have also identified differential alternative splicing and expression of novel exons in both cell types. In particular, differences in alternative splicing in 31 genes and expression of 19 novel exons in 12 different genes in TSCs and TGCs were identified. These findings will enhance the understanding of placental development in mammals at the molecular level and provide a useful resource to the scientific community interested in studying placental biology.

## Methods

### TSC culture and differentiation into TGCs

TSCs isolated from C57BL/6 laboratory mice were cultured in PMEF-conditioned medium (RPMI) supplemented with 10% ES-FBS (Invitrogen), 1% Antibiotic-Antimycotic (Invitrogen), 25 ng/ml FGF4 (Sigma), 1 μg/ml Heparin (Sigma), 0.1 mM 2-Mercaptoethanol (Sigma), and 1 mM sodium pyruvate (Sigma) at 37 °C in a humidified chamber with 5% CO_2_ as described elsewhere [15, 22]. The differentiation of TSCs into polyploid TGCs was induced by growing the cells in RPMI medium supplemented only with 10% FBS and 1% Antibiotic-Antimycotic. For the best differentiation, TSCs were kept in differentiation medium for 4 days. For subculturing, TSCs were detached from the plates with 0.05% trypsin (Sigma-Aldrich) for 5–10 min. Trypsin was inactivated by addition of culture medium to the flasks; cells were centrifuged and resuspended in culture medium and replated at the desired density.

### Immunofluorescence staining and confocal microscopy

TSCs were grown on coverslips in 6-well plates at a density of 0.05 million for undifferentiated control cells and 0.5 million for differentiation into TGCs or for treatment with Aurora A inhibitor. Cells on coverslips were washed with PBS and fixed with 4% formaldehyde for 15 min at room temperature. The cells were then washed again with PBS, followed by permeabilization and blocking with 0.1% Triton X solution prepared in 2% BSA for 20 min at room temperature. After permeabilization, cells were washed with PBS and incubated with phalloidin Texas Red (Invitrogen) or phalloidin Alexa Fluor 488 (Thermofisher Scientific) or α-tubulin antibody (Abcam, 1:500 dilution) for an hour. For tubulin staining, cells were washed and incubated for another hour with AlexaFluor 488-labeled secondary antibody. The coverslips for both phalloidin and tubulin staining were washed five times with PBS, stained with DAPI (in the penultimate wash), and mounted on the slides using Fluoromount™. Images were taken using a confocal microscope (Nikon Model C2+).

### Flow cytometry

For FACS analysis, TSCs were cultured in T-25 flasks at a density of 0.1 million for undifferentiated control cells and 1 million for differentiation into TGCs. Cells were collected by trypsinization, washed with 1X PBS (containing 1% FBS), and fixed with 75% ice-cold ethanol. Fixed cells were treated with 0.5% RNase A (Invitrogen) to digest RNA and stained with propidium iodide solution (10 μg/ml, Invitrogen) for 30 min at 37 °C. The samples were then analyzed on BD FACS Calibur (Becton Dickinson).

### Total RNA isolation for global transcriptome analysis

For differential gene expression analysis, TSCs were cultured at a density of 0.2 million for undifferentiated control cells and 2 million for differentiation into TGCs cells in T75 flasks for 4 days. Cells were trypsinized and harvested by centrifugation at 1500 rpm and washed with PBS. Total RNA was extracted from 2 million TSCs and TGCs in three independent experiments using RNeasy Plus Mini Kit (Qiagen) according to the manufacturer’s protocol. The quality of RNA was measured by running the total RNA on gels and by measuring the 260/280 and 260/230 absorbance ratios. Once the quality of the samples and differentiation were confirmed, samples were shipped on dry ice to BGI Genomics, Hong Kong. Four gigabases of RNA-Seq data was received for each sample (Table S1). The quality controls for each of the six samples were also obtained.

### Bioinformatics analysis of RNA-Seq data

RNA-Seq data of TSCs and TGCs was aligned to mouse reference genome MmGRCm38 downloaded from Ensembl using TopHat for downstream analysis. Bioinformatics analysis was performed for the detection of differentially expressed genes and differentially expressed exons, and for the identification of novel exons. Analysis of differential gene expression analysis was carried out using a python script and R packages. The read sequences in the BAM file were first converted into count tables by htseq-count [23]. The resulting tables were then processed by two R packages (EdgeR and DESeq2) for the identification of differentially expressed genes [24, 25]. The differential expression (upregulation or downregulation) was calculated as fold change and presented as log2 of the fold change (Additional files 1 and 2). Differential expression of exons relative to the overall expression of the respective genes was detected using JunctionSeq, a Bioconductor package used for differential exon and splice-junction operation in transcriptome data [26]. For the detection of novel exons/genes, we used AUGUSTUS, a web-based server for prediction of unannotated exons [27]. Exons that did not overlap with any of the known exons of respective genes in the Ensembl or GENCODE comprehensive transcript database were considered to be novel.

### Classification and ontology of differentially expressed genes

Differentially expressed genes were manually grouped into various functionally distinct protein families (genes with > 2-fold difference). For classification into signaling pathways, the Protein Analysis Through Evolutionary Relationships (PANTHER v.15.0) Classification System was used [28]. The gene ontology (GO) analysis of differentially expressed genes was performed in the categories of biological process, molecular function, and cellular component using gene ontology resource [29, 30].

### Experimental validation of differentially expressed genes and exons

Validation of differential expression of the identified genes and exons was carried out through real-time PCR with cDNA made from TSCs and TGCs. To avoid any traces of DNA in the total RNA, samples were DNase treated using Turbo DNase Kit (Ambion, USA). DNase was later inactivated according to the kit protocol. First-strand synthesis was carried out with SuperScript III RT Kit (Invitrogen) and oligodT, in accordance with the manufacturer’s protocol. Quantitative PCR was then performed to quantify the mRNA levels of differentially regulated genes and exons. Actin was used as an internal control for all the experiments. The primers were designed with Primer-Blast and synthesized by Macrogen, Korea (Table S5) [31]. The PCR reactions (20 μl each; performed in triplicate) contained 10 μl of 2X SYBER Green PCR Master Mix (Life Technologies, UK), 0.2 μl each of the forward (20 pmol) and reverse (20 pmol) primers, and 100 ng cDNA template. The qPCR was performed on Applied Biosystem 7500/7500 Fast Real-Time PCR system (CA, USA). Thermal cycling was performed using machine pre-set two-step cycling protocol. It consisted of 95 °C for 10 min, followed by 40 cycles of 95 °C for 15 s and 60 °C for 1 min.

Data were collected and analyzed by the 7500 real-time PCR System analysis software and exported to Microsoft Excel for final calculations. We used a comparative CT (threshold cycle) to calculate the relative quantification of gene expression.

ΔCT = CT target − CT reference.

where CT reference is the mean value of Actin and CT target is the CT-mean value for the investigated gene (each sample was run in triplets).

ΔΔCT = ΔCT test sample − ΔCT calibrator sample.

### Statistical analysis

The statistical analysis was performed using GraphPad Prism. Two-sided, unpaired *t* test was performed for each gene and *P* values < 0.05 were deemed significant. The level of significance is shown using asterisk (*). *P < 0.05, **P < 0.01, ***P < 0.001, and ****P < 0.0001.

### Experimental validation of novel exons

For validation of novel exons, RNA samples and cDNA were prepared as described above. cDNA was subjected to polymerase chain reaction (PCR) amplification using primers targeting novel exons. Each reaction mixture of 20 μl contained 100 ng template cDNA, 0.2 μl of each forward (20 pmol) and reverse (20 pmol) primers, 2 μl 10 X PCR buffer with (NH_4_)_2_SO_4_, 2 μl 25 mM MgCl_2_, 0.2 μl of 0.5 unit Taq DNA polymerase (Thermo Scientific), 1 μl 10 mM dNTPs (Invitrogen), and 13.4 μl PCR water. PCR was carried out in Veriti 96-Well Thermal Cycler (Thermo Fisher Scientific). Amplification was carried out using initial denaturation at 95 °C for 5 min followed by 40 cycles of 95 °C for 1 min, 60 °C for 20–30 s, 72 °C for 20–40 s, and a final extension at 72 °C for 7 min. The PCR product was subsequently resolved on 2% agarose gel and images were taken using ChemiDoc system (BioRad).

### Immunoblotting

Immunoblotting was performed from 4 days undifferentiated TSCs and differentiated TGCs as described previously [32]. Briefly, cells were lysed with Triton lysis buffer (50 mM NaCl, 20 mM Tris pH 7.5, 1 mM EDTA, 1% Triton X100, 50 mM NaF, protease inhibitors and phosphatase inhibitors) on ice. Cell lysates were cleared by centrifugation and protein concentration was measured by Bradford reagent. Equal amounts of proteins were resolved by SDS-PAGE and transferred onto nitrocellulose membranes. Membranes were blocked and incubated overnight with primary antibodies (Aurora A; Cell Signaling, Borealin; Novus Biologicals and Tubulin; Santa Cruz). The next day, membranes were washed and incubated with HRP-labeled anti-mouse or anti-rabbit (Abcam) antibodies for 1 h at room temperature. Membranes were washed and developed with ECL on BioRad Chemidoc.

## Results

### Differentiation of trophoblast stem cells into trophoblast giant cells

**Fig. 1:**
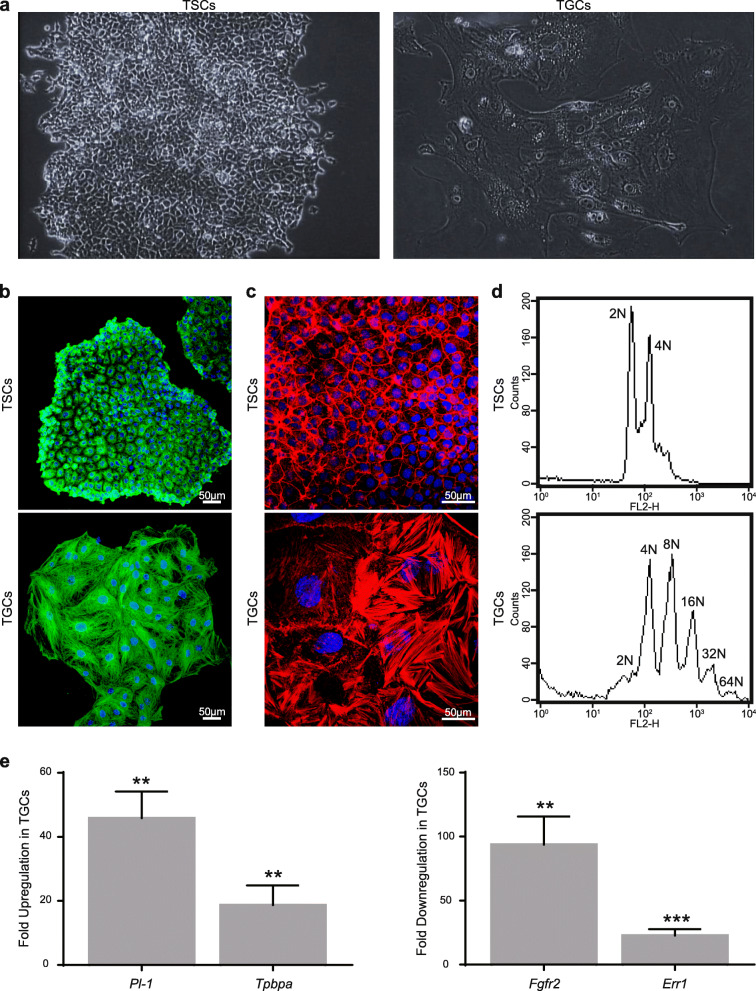
Differentiation and characterization of mouse trophoblast cells. **a** Representative bright-field images of undifferentiated and differentiated trophoblast cells. **b** Immunofluorescence imaging of TSCs and TGCs stained with alpha-tubulin. Cells were fixed with paraformaldehyde and stained with alpha-tubulin and Alexa Flour 488-conjugated secondary antibodies. DAPI was used for nuclear (DNA) staining (blue). **c** Immunofluorescence imaging of TSCs and TGCs for actin. Cells were fixed with paraformaldehyde and stained with Texas red phalloidin and DAPI. **d** Induction of polyploidy in differentiated TGCs. TSCs and TGCs were fixed, stained with propidium iodide, and analyzed through FACS. **e** mRNA expression analysis of TSC and TGC specific markers before and after differentiation. Expression levels of TGC-specific markers, *Pl-1* and *Tpbpa* (*left*), and TSC-specific markers *Err1* and *Fgfr2* (*right*) were analyzed through real-time PCR. Error bars represent SEM for 3 independent biological replicates.

Trophoblast stem cells with diploid genomes are smaller in size and form compact colonies. Once the differentiation of TSCs was induced by removing FGF4 from the medium, the cells became progressively larger with very heterogeneous shape, size, and ploidy level (Fig. 1), as described previously [15]. For RNA-Seq analysis, both TSCs and TGCs populations were confirmed according to various literature-based cytological and molecular criteria [7, 15, 33] (Fig. 1a–d). Morphological changes associated with the differentiation of TSCs to TGCs, including increased cell size and rearrangement of actin and microtubule cytoskeletons, were seen in TGCs (Fig. 1a–c). Similarly, TGCs were polyploid with multiple copies of genome ranging from 4N to as high as 64N [34] (Fig. 1d). At the molecular level, the expression of TGC-specific markers, placental lactogen-1 (*Pl-1*), and trophoblast-specific protein alpha (*Tpbpa*) were markedly upregulated in TGCs as compared to TSCs. Conversely, TSC-specific markers, estrogen-related receptor alpha (*Err1*) and fibroblast growth factor receptor 2 (*Fgfr2*), were downregulated in TGCs compared to TSCs (Fig. 1e). All these data confirm that we had a bona fide population of TSCs and TGCs for our subsequent experiments.

### Genome-wide differential gene expression in TSCs and TGCs

**Fig. 2:**
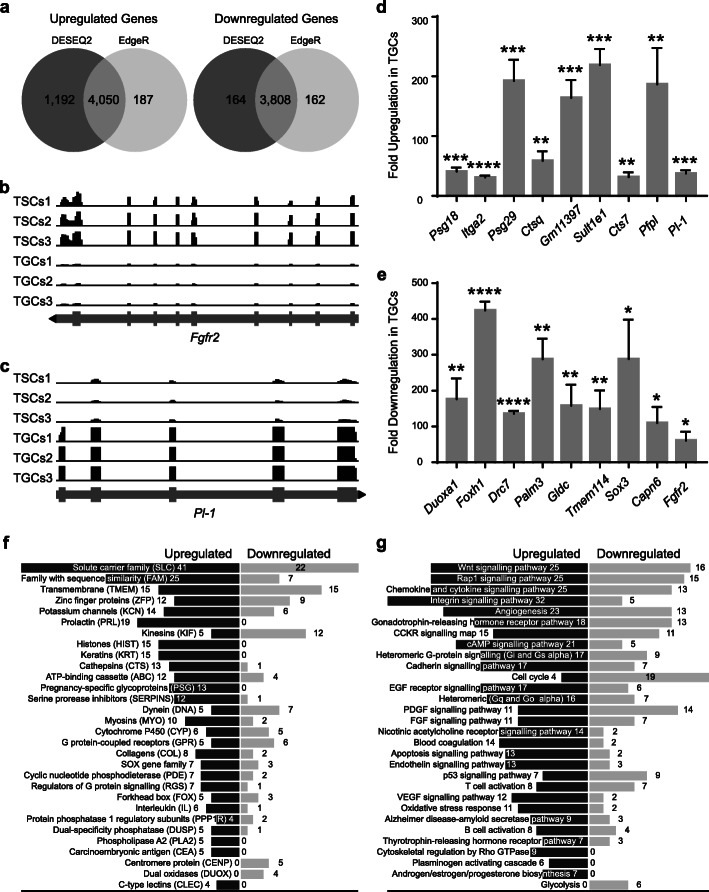
Analysis of differentially expressed genes in TSCs and TGCs. **a** Venn diagrams showing the number of differentially expressed upregulated (*left*) and downregulated (*right*) genes in TSCs and TGCs identified through DESEQ2 (*dark gray*), EdgeR (*light gray*), and both (*overlap*) programs. **b** Integrated genome viewer (IGV) snapshot of the *Fgfr2* locus illustrating read coverage in all 3 replicates of TSCs and TGCs. Reference gene track is shown at the bottom (*gray line*) with bars representing the corresponding exons and arrowhead (*black*) showing gene orientation. **c** IGV snapshot displaying the read coverage within the *Pl-1* gene, a known marker of TGCs, in all 3 replicates of undifferentiated and differentiated cells*.***d** mRNA expression analysis of 8 selected upregulated genes using real-time PCR. Expression of *Pl-1* was used as a marker for TGCs. **e** mRNA expression analysis of 8 selected downregulated genes identified through real-time PCR. Amplification of *Fgfr2* was used as a known marker of TSCs. Error bars represent SEM of 3 independent biological replicates. **f** Classification of differentially expressed genes to functionally distinct classes of protein families. **g** PANTHER pathway enrichment of differentially expressed genes in TGCs.

Significant changes in the expression of 9214 and 8207 genes were identified using DESeq2 and EdgeR, respectively, with a false discovery rate of < 0.05 (7858 overlapping genes identified by both the packages; Fig. S1). These represent approximately 20% and 18% of the total genes in the mouse genome with nucleotide sequence data [35]. Among the genes downregulated in TGCs, a total of 3972 and 3970 genes were identified using DESeq2 and EdgeR, respectively, with 3808 overlapping genes. Similarly, the number of upregulated genes identified by DESeq2 and EdgeR were 5242 and 4237, respectively, with 4050 genes identified by both R packages (Fig. 2a). Several genes known to be differentially regulated in TGCs were identified in our analysis (Table S2) thereby validating both the differentiation of TSCs and the analysis of differential gene expression. Read coverages for fibroblast growth factor receptor 2 (*Fgfr2*) and placental lactogen-1 (*Pl-1*) are shown as examples of genes downregulated and upregulated in TGCs, respectively (Fig. 2b, c).

### Validation of differentially regulated genes

Next, we validated the expression of some of the top differentially regulated genes through real-time PCR. Eight different genes from each of the top 15 upregulated and downregulated genes in TGCs were analyzed. The expression of *Psg18*, *Itga2*, *Psg29*, *Ctsq*, *Gm11397*, *Sult1e1*, *Cts7*, and *Pfpl* was significantly upregulated in the differentiated TGCs (Fig. 2d), whereas the expression of *Duoxa1*, *Foxh1*, *Drc7*, *Palm3*, *Gldc*, *Tmem114*, *Sox3*, and *Capn6* was significantly downregulated following differentiation (Fig. 2e). The cell-type specific expression levels of *Fgfr2* and *Pl-1* were used as a TSC- and TGC-specific marker, respectively. Confirmation of these genes through real-time PCR and the reproduction of the expression pattern of cell-type-specific markers further validated the reliability of our RNA-seq data.

### Classification of the differentially expressed genes

Analysis of differential expression (at least 2-fold difference) of genes encoding functionally distinct protein families revealed solute carrier family (SLC) proteins to be the most affected with 41 upregulated and 22 downregulated genes in TGCs (Fig. 2f). The next largest group of proteins was the family with sequence similarity (FAM; 25 upregulated and 7 downregulated genes) followed by transmembrane (TMEM) and zinc finger proteins (ZFP) families. A large number of genes encoding for prolactins (PRL), histones (HIST), keratins (KRT), and pregnancy-specific glycoproteins (PSG) were exclusively upregulated in TGCs. No genes encoding members of these protein families were downregulated, implicating their TGC-specific roles (Fig. 2f). Regulated expression of proteins belonging to these groups is critical for the normal function of TGCs and healthy outcome of pregnancy. Targeted deletion of type I keratins, K18 and K19 (2.33- and 3-fold increase in TGCs) in mice, for example, results in fragile TGCs that cause embryonic lethality [36]. Similarly, the lethality of K8 knockout (type II keratin with 3-fold increase in TGCs) embryos results from failure of TGCs barrier function [37]. Other keratins with even higher expression in TGCs include K13 (9-fold), K14 (7.2-fold), K36 (6.6-fold), K37 (5-fold), K25 (4-fold), K16 (4.15-fold), and K15 (4.11-fold). Whether these keratins are also as critical in TGC function and embryonic development remains to be determined.

Differentiation of mouse TSCs into TGCs is associated with changes in activities of different cellular pathways and increased ploidy level. Grouping of differentially expressed genes (at least 2-fold change) according to their roles in various pathways revealed almost exclusive expression of components of some of the key cellular pathways in one or the other cell type (Fig. 2g). Genes encoding components of integrin signaling, for example, were overwhelmingly upregulated in TGCs (32 upregulated versus 5 downregulated genes). Expression of genes involved in cytoskeletal regulation by Rho GTPase, plasminogen activating cascade, and androgen/estrogen/progesterone biosynthesis was exclusively upregulated in TGCs (9, 6, and 7 upregulated genes respectively, versus downregulation of none). Activation of pathways in trophoblast cells involving these upregulated genes is critical for the successful blastocyst invasion and implantation into the endometrium [38–40]. Regulated expression of integrins, for example, is critical for implantation; anomalies in integrin expression, therefore, can result in placental diseases including preeclampsia [41]. Some other important pathways that showed domination of upregulated genes in TGCs include cAMP signaling, cadherin signaling, EGF receptor signaling, nicotine acetylcholine receptor signaling, blood coagulation, endothelin signaling, VEGF signaling, and oxidative stress response. On the contrary, genes involved in the glycolytic pathway and cell cycle regulation were exclusively or mostly downregulated in TGCs indicating their trophoblast stem cell-specific functions (Fig. 2g).

### Gene ontology enrichment analysis of differentially expressed genes

To determine the functionality of differentially expressed genes (with at least 2-fold change), gene ontology (GO) analysis was performed. Significantly enriched GO terms (Fisher’s exact test, FDR-adjusted *p* value < 0.05) were obtained from the enrichment of differentially expressed genes into biological process, molecular function, and cellular component categories (Additional files 3, 4, 5, 6, 7 and 8). The total number of GO terms obtained for upregulated and downregulated differentially expressed genes were 748 and 849, respectively. For the upregulated genes, significantly enriched GO terms (classified by fold enrichment value) in the category of biological process were regulation of lactation, TGC differentiation, cellular response to VEGF stimulus, maternal placenta development, and female pregnancy (Fig. S2A, Additional file 3). The top significantly enriched GO terms in the category of molecular function were prolactin receptor binding, cell adhesion mediator activity, and calcium-dependent phospholipid binding (Fig. S2B, Additional file 4). The cellular component category included integrin complex, euchromatin, and protein complexes involved in cell adhesion (Fig. S2C, Additional file 5). The enrichment of GO terms for upregulated genes, therefore, reflects processes, molecular functions, and cellular components that are involved in TGC function and implantation.

For the downregulated genes, the top significantly enriched GO terms in the biological process category were centrosome separation, regulation of smooth muscle cell chemotaxis, and regulation of attachment of spindle microtubules to the kinetochore (Fig. S2A, Additional file 6); the molecular function category included DNA replication origin binding, damaged DNA binding, and DNA-dependent ATPase activity (Fig. S2B, Additional file 7) and for the cellular component category, Ndc80 complex, NMS complex, and chromosome passenger complex were present (Fig. S2C, Additional file 8). Enrichment of most of these terms indicates reduced processes, molecular functions, and cellular components involved in cell division.

### Validation of Aurora A kinase as a regulator of mouse TSC differentiation

**Fig. 3:**
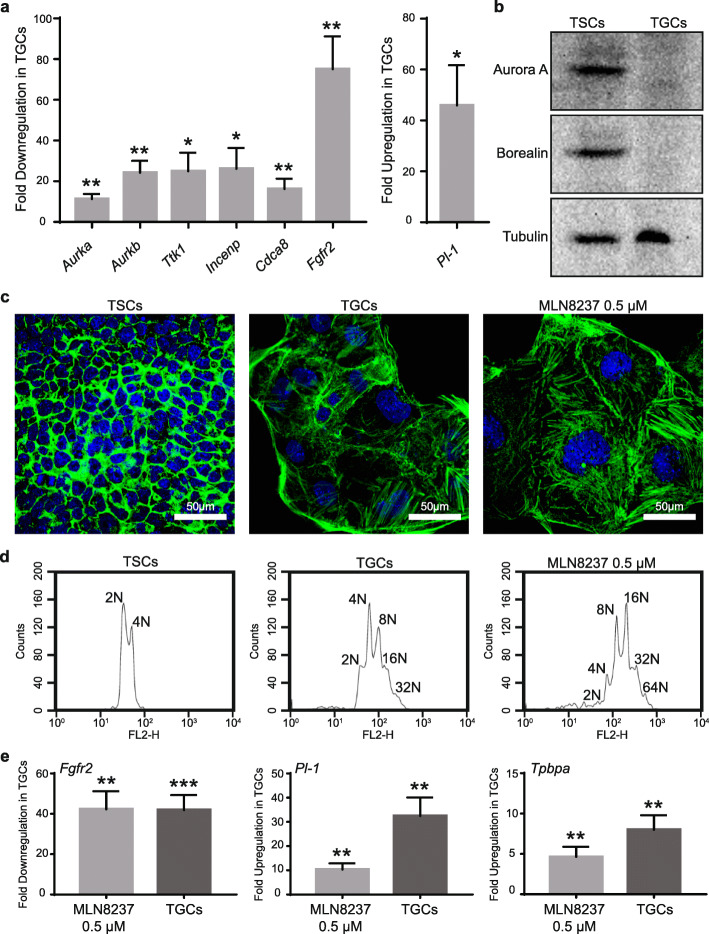
Analysis of downregulated CPC complex genes in TSCs and TGCs. **a** mRNA expression analysis of 5 selected downregulated mitotic genes using real-time PCR. Error bars represent SEM of 3 independent biological replicates. **b** Western blot analysis of Aurora A and Borealin in TSCs and TGCs differentiated for 4 days. **c** Immunofluorescence imaging of solvent control-treated TSCs, TSCs treated with Aurora A inhibitor MLN8237, and TGCs for actin and nucleus. Cells were fixed with paraformaldehyde and stained with phalloidin Alexa Fluor 488 and DAPI. **d** FACS analysis of TSCs, TSCs treated with Aurora A inhibitor MLN8237, and TGCs. **e** mRNA expression analysis of *Fgfr2*, *Pl-1*, and *Tpbpa* in TSCs, TGCs, and TSCs treated with MLN8237 for 3 days. Error bars represent SEM of 3 independent biological replicates.

For functional validation of the RNA-seq data, we searched for differentially expressed regulators of the cell cycle that may become redundant for the non-dividing TGCs. Five different mitotic genes (*Aurka*, *Aurkb*, *Ttk1*, *Incenp*, and *Cdca8*) were selected for validation through real-time PCR. The proteins encoded by these genes are involved in initiation and progression through mitosis and include components of the chromosomal passenger complex (CPC). As expected, the expression of all five selected genes, *Aurka*, *Aurkb*, *Ttk1*, *Incenp*, and *Cdca8* was significantly downregulated in the differentiated TGCs (Fig. 3a). Furthermore, the expression of Aurora A (*Aurka*) and Borealin (*Cdca8*) proteins was also significantly reduced in TGCs compared to TSCs (Fig. 3b).

Aurora A kinase is required for embryonic stem cell (ESC) self-renewal and pluripotency and its downregulation leads to differentiation of ESCs [42]. Reduced Aurora A expression in TGCs prompted us to hypothesize that Aurora A may also be required for self-renewal of TSCs and its inhibition may result in their differentiation into TGCs. We, therefore, probed the role of Aurora A in the differentiation of TSCs through inhibition of Aurora A activity. Treatment of TSCs for 3 days with Aurora A-specific inhibitor, MLN8237 (0.5 μM), induced differentiation in the presence of FGF4 as demonstrated by the increase in cell and nuclear size as well as induction of polyploidy (Fig. 3c, d). At the molecular level, MLN8237-induced differentiation was confirmed through a significant increase in expression of TGC-specific markers, *Pl-1* and *Tpbpa*, and decrease in expression of TSC-specific marker *Fgfr2* in TGCs (Fig. 3e). Results from a phenotypic chemical genetic screen in our lab show that several Aurora A-specific (MLN8237, MLN8054, and MK-8745), Aurora B-specific (AZD1152 and GSK1070916), and pan-Aurora inhibitors (VX-680, PHA739358, CCT137690, AMG-900, SNS-314, and CYC116) induce TSC differentiation as determined by the increased nuclear size (Table S3 and Fig. S3; manuscript for the complete screen in preparation). A similar kinase inhibitor screen recently identified that several Aurora kinase inhibitors (including CCT137690, MLN8237, AT9283, and TC-A-2317) reduce CDX2 (a stem cell marker) expression in TSCs, thereby inducing their differentiation [43]. Thus, we conclude that Aurora A is required for maintaining the stemness of TSCs and inhibition of its activity results in their differentiation.

### Differential expression of exons in TSCs and TGCs

**Fig. 4:**
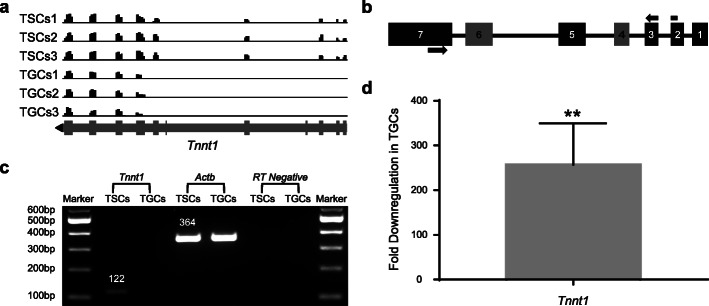
Experimental validation of differentially expressed exons in *Tnnt1* gene. **a** IGV snapshot of differentially expressed exons identified in *Tnnt1* gene. Read coverage of differentially expressed exons 1, 2, 3, 5, and 7 in *Tnnt1* gene are shown in all 3 replicates of TSCs and TGCs. Reference gene track is shown at the bottom (*gray line*) with bars representing the corresponding exons and arrowhead (*black*) showing the orientation of the gene. **b** Primer design strategy used for the amplification of differentially expressed exons in *Tnnt1* gene. Boxes represent differentially expressed exons (*black*) in TSCs and exons with no expression (*dark gray*) in both TSCs and TGCs. Forward primer (*top arrow*) spanned exons 2 and 3 while reverse primer (*bottom arrow*) was in exon 7. Connecting line (*black*) between the boxes represents the intronic region. **c** Differential amplification of *Tnnt1* gene in TSCs and TGCs. Specific exons in *Tnnt1* gene and actin were amplified from TSCs and TGCs through PCR. Gel image of the PCR-amplified products of *Tnnt1* gene and the positive control (actin) from TSCs and TGCs is shown. PCR amplification from RT-negative (without reverse transcriptase) samples with actin primers represents the negative control. **d** mRNA expression analysis of differentially expressed exons in *Tnnt1* gene by quantitative real-time PCR. Error bars represent SEM of 3 independent biological replicates.

Transcript variants resulting from differential expression of exon(s) may play a role in trophoblast stem cell differentiation or function by encoding functionally distinct protein isoforms in TSCs and TGCs. We, therefore, analyzed the RNA-seq data for transcript variants and identified that exons in 31 genes were differentially expressed in these two cell types (Methods; Table 1). Specific expression of one or more exons in 15 genes was seen in TSCs while one or more exon(s) in 16 other genes were found to be specifically expressed in TGCs (Table 1). Read coverage of five different exons (exons 1, 2, 3, 5, and 7) in *Tnnt1* gene, for example, was seen only in TSCs (Fig. 4). Exons 4 and 6, on the contrary, had no read coverage in both TSCs and TGCs (Fig. 4a). TSC-specific expression of exons in the *Tnnt1* gene was confirmed by PCR amplification of cDNA region covering exons 2 to 7 (122 bp) using specific primers (Fig. 4b, c). Moreover, the amplification of TSC-specific exons of *Tnnt1* gene using real-time PCR showed significant downregulation of expression in TGCs (*P* ≤ 0.01; Fig. 4d), thereby validating their differential expression in the two cell types. Similar validation of cell type-specific expression of exons in nine different genes was done through cDNA amplification from both cell types with real-time PCR (Fig. S4). The expression of specific exons (Table 1) in *Garnl3*, *Lrrfip1*, *Lmo7*, *Dnmbp*, *Kalrn*, and *Asic1* genes was significantly downregulated whereas the expression of some exons in *Tmem40*, *P2ry2*, and *Ptprk* genes was significantly upregulated in TGCs compared to TSCs (Fig. S4b and c). The differential expression of *Pl-1* and *Fgfr2* in the same experiment confirmed the differentiation at the molecular level (Fig. S4b and c).

**Table 1:** Genes with differentially expressed exons in TSCs (15 genes) or TGCs (16 genes) are shown. The number of differentially expressed exons and the respective gene loci are also shown

Gene name	Description	(Exon)s	Expressed in	Locus
*Tnnt1*	Troponin T1, skeletal, slow	1, 2, 3, 5, 7	TSCs	7:4,494,599-4,526,352
*Garnl3*	GTPase-activating Rap/Ran-GAP domain-like	12	TSCs	2:33,026,380-33,036,319
*Lmo7*	LIM domain only 7	2	TSCs	14:101,793,612-101,795,315
*Dnmbp*	Dynamin-binding protein	1	TSCs	19:43,846,821-43,940,191
*Kalrn*	Kalirin RhoGEF kinase	12, 13	TSCs	16:33,966,718-34,009,600
*Asic1*	Acid-sensing (proton-gated) ion channel 1	6–8	TSCs	15:99,678,919-99,709,681
*Lrrfip1*	Leucine rich repeat (in FLII) interacting protein 1	11, 20	TSCs	1:91,122,108-91,124,774
*Kif7*	Kinesin family member 7	13	TSCs	7:79,696,662-79,714,284
*Ceacam19*	Carcinoembryonic antigen-related cell adhesion molecule 19	2–5	TSCs	7:19,877,891-19,890,114
*Nlrp4c*	NLR family, pyrin domain containing 4C	2, 3	TSCs	7:6,049,504-6,081,257
*Mybpc*	Myosin-binding protein C, cardiac	4, 5	TSCs	2:91,118,144-91,136,516
*Pamr1*	Peptidase domain containing associated with muscle regeneration 1	3–5	TSCs	2:102,550,012-102,643,041
*Tcp11l1*	T-complex 11-like protein 1	1–7	TSCs	2:104,678,017-104,712,156
*Nphp1*	Nephronophthisis 1 (juvenile) homolog (human)	12–15	TSCs	2:127,740,732-127,788,897
*Hbp1*	High mobility group box transcription factor 1	1	TSCs	12:31,926,254-31,950,535
*Tmem40*	Transmembrane protein 40	1	TGCs	6:115,758,238-115,760,280
*P2ry2*	Purinergic receptor P2Y, G-protein coupled 2	1	TGCs	7:100,996,568-101,012,866
*Ptprk*	Protein tyrosine phosphatase receptor type K	16	TGCs	10:28,560,239-28,563,804
*Sult1e1*	Sulfotransferase family 1E, member 1	5, 6	TGCs	5:87,575,600-87,592,174
*Cirbp*	Cold-inducible RNA-binding protein	1	TGCs	10:80,165,620-80,167,402
*Chmp6*	Charged multivesicular body protein 6	5	TGCs	11:119,913,441-119,919,548
*Scd3*	Stearoyl-coenzyme A desaturase 3	1	TGCs	19:44,203,288-44,244,016
*Rlbp1*	Retinaldehyde-binding protein 1	4	TGCs	7:79,379,374-79,391,552
*Fanci*	Fanconi anemia, complementation group 1	1	TGCs	7:79,388,339-79,404,215
*Slc1a2*	Solute carrier family 1 (glial high affinity glutamate transporter), member 2	2	TGCs	2:102,658,659-102,790,784
*Cd44*	CD44 antigen	4	TGCs	2:102,900,136-102,939,896
*Ehf*	ETS homologous factor	6, 7	TGCs	2:103,272,963-103,275,447
*Ccdc73*	Coiled-coil domain-containing protein 73	8	TGCs	2:105,011,117-105,021,056
*Gpat2*	Glycerol-3-phosphate acyltransferase 2, mitochondrial	8–14	TGCs	2:127,424,729-127,435,622
*Fermt1*	Fermitin family homolog 1	3	TGCs	2:132,904,389-132,945,906
*Acan*	Aggrecan core protein	1–7	TGCs	7:79,090,765-79,122,518

### Identification of novel exons in trophoblast cells

**Fig. 5:**
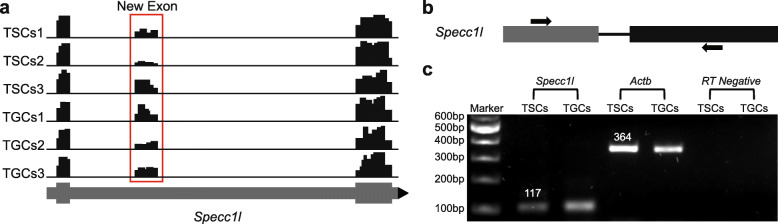
Experimental validation of novel exon identified in *Specc1l* gene. **a** IGV snapshot of the novel (unannotated) exon identified in *Specc1l* gene illustrating read coverage in all 3 replicates of both TSCs and TGCs cells. Read coverage for novel exon is enclosed by a red box. Reference gene track is shown at the bottom (*gray line*) with bars representing the corresponding exons and arrowhead (*black*) showing the orientation of the gene. **b** Primer design strategy used for the amplification of novel exon. Forward primer (*top arrow*) was designed in a known exon (*dark gray rectangle*) while reverse primer (*bottom arrow*) was designed in a novel exon (*black rectangle*). The two exons are separated by the intronic region (*black line*). **c** PCR amplification of the novel exon from TSCs and TGCs. Agarose gel image of the PCR-amplified products of a novel exon in *Specc1l* gene. Amplification of actin (*Actb*) from RT+ and RT− (with and without reverse transcriptase) represents positive and negative controls, respectively.

Due to the paucity of available data for trophoblast cells at a single base resolution, we also sought to identify the presence of novel exons in the mouse genome using the RNA-seq data. We found a total of 19 novel exons in 12 different genes for which no annotation was present in the Ensembl genome or GENCODE Comprehensive database (Methods; Table 2). The expression of ten of these novel exons was validated through PCR amplification of cDNA prepared from TSCs and TGCs (Fig. 5 and S5). Most of the novel exons had read coverage over the mapped region of the mouse genome in both undifferentiated TSCs and differentiated TGCs suggesting that expression of these exons might be trophectoderm specific. The novel exon detected in *Specc1l* gene, for example, had read coverage in all the replicates of both TSCs and TGCs (Fig. 5a).

**Table 2:** Genomic location, number of novel exon(s) identified in each gene, and number of novel predicted gene models are shown in the table

Gene	Description	Genomic location	Expressed in	No of novel exons	Predicted novel gene models
*Asprv1*	Aspartic peptidase, retroviral-like 1	6:86625226-86625374 6:86626038-86626286 6:86627771-86627914	TSCs/TGCs	3	1
*Zbtb7c*	Zinc finger and BTB containing 7C	18:75894667-75894754 18:76095436-76095475	TSCs/TGCs	2	2
*Sil1*	Endoplasmic reticulum chaperone SIL1 homolog (*S*. *cerevisiae*)	18:35527540-35527984	TGCs	1	1
Ai506816	Expressed sequence AI506816	5: 23692428-23695320	TSCs/TGCs	1	1
*Specc1l*	Sperm antigen with calponin homology and coiled-coil domains 1	10:75274671-75274823	TSCs/TGCs	1	1
*Map2k2*	Mitogen-activated protein kinase kinase 2	10:81125447-81125546 10:81128227-81128323	TSCs/TGCs	2	1
*Snx29*	Sorting nexin 29	16:11429104-11421935 16:11729997-11730136	TSCs/TGCs	2	1
*Zdhhc23*	Zinc finger, DHHC domain containing 23	16:43979457-43979753	TSCs/TGCs	1	1
*Gramd1c*	GRAM domain containing 1C	16:44016088-44016108 16:44001636-44001788	TSCs/TGCs	2	2
*Angptl6*	Angiopoietin-like 6	9:20877287-20877320	TSCs/TGCs	1	1
*Adrb3*	Adrenergic receptor, beta 3	8:27235582-27235647 8:27243806-27243974	TSCs/TGCs	2	1
*Eps8l1*	EPS8-like 1	7:4475109-4475152	TSCs/TGCs	1	1

On the other hand, novel exon identified in the *sil1* gene showed read coverage only in the three replicates of TGCs (Fig. S5a). For PCR amplification of the novel exons from the cDNA, two different strategies were used for primer designing. Some exons were validated using forward and reverse primer designed in different exons while others were validated using the primer pairs designed within a single novel exon (Fig. S5b). For validation of the novel exon identified in *Specc1l* gene, forward and reverse primers were designed in the reported and the newly identified exons, respectively (Fig. 5b). The resulting amplification of a 117 bp PCR product confirmed the expression of new exon and that no intronic region was amplified (Fig. 5c). The PCR amplification using specific primers also validated the TGC-specific expression of novel exon detected in the *Sil1* gene in accordance with the read coverage data of IGV. The read coverage, primer designs, and PCR amplification for eight more genes are shown in Supplementary Fig. 5. Amplification of actin with reverse and forward primers in two different exons (364 bp product size without introns) ruled out any false positives from the genomic DNA contamination in cDNA from both TSCs and TGCs. Finally, new gene models predicted by AUGUSTUS [27] were built for all the genes in which novel exons were identified (Fig. S6).

## Discussion

We have identified a large number of differentially expressed genes in TSCs and TGCs, including many genes that have not been previously implicated in TSC differentiation or TGC function. We also discovered differential alternative splicing and expression of novel exons in the two cell types which may be specific for the trophectoderm lineage. Genes encoding components of various signaling pathways and cellular processes were among those that were identified to be differentially regulated in the two cell types. For example, mitotic proteins Aurora A kinase (*Aurka*) and components of the chromosomal passenger complex (CPC), *Aurkb*, *Ttk1*, *Incenp*, *Cdca8*, and *Birc5* were significantly downregulated in TGCs. Since Aurora A and CPC complex regulate key mitotic events, from mitotic entry to chromosome alignment and cytokinesis [44], their downregulation may be responsible for preventing mitotic cell division and promoting differentiation in TSCs. Indeed, the inhibition of Aurora kinases with small-molecule inhibitors targeting only Aurora A (MLN8237) or both Aurora A and B (CCT137690) induced differentiation of TSCs (Fig. 3, Table S3 and Fig. S3). A recent kinase inhibitor screen for inducers of TSC differentiation also identified Aurora kinase inhibitors including CCT137690 [43]. CDK1 activity is required for activation of Aurora A during G2/M transition [45]. Unlike mitotic cells, TGCs do not undergo G2/M transition and do not have CDK1 activity [15]. Thus, it is quite conceivable that TGCs will not need Aurora A activity and expression. In total, 19 genes involved in cell cycle regulation were downregulated in TGCs compared to only 4 that were upregulated. Two of the upregulated genes involved in the cell cycle are *Ccne2* and *Cdk6*. During differentiation of TSCs, *Ccne2* and *Cdk6* might facilitate the transition into S phase and assembly of pre-replication complexes on DNA for re-replication [46, 47]. This finding is in agreement with recent gene expression profiling of human trophoblast differentiation using microarrays, where several cell cycle-related genes were downregulated in syncytiotrophoblasts [48] and supports the observation that TGCs do not undergo a conventional mitotic cell cycle. Some of the genes (e.g., *Cdc6*, *Cdc20*, and *Ccnb2*) were downregulated in both systems.

*Sox3*, a member of the SOX family of transcription factors that make cell fate decisions during development [49], was downregulated in TGCs. Since TGCs are terminally differentiated, downregulation of *Sox3* will be a logical step for them. Previous studies have identified that *Sox21*, another downregulated SOX family member in TGCs, is highly expressed in TSCs and is implicated in their differentiation into TGCs [50]. Another transcription factor, Stox1, was upregulated in TGCs (2.93-fold increase); its loss of function is associated with preeclampsia in a large cohort of Dutch females indicating the significance of the elevated expression in TGCs [51]. Six genes involved in the glycolytic pathway were downregulated in TGCs compared to none in the TSCs; this corroborates the reported higher metabolic activity of undifferentiated cytotrophoblasts as compared to differentiated syncytiotrophoblasts [52]. Similarly, FGF4 has been shown to maintain stemness in TSCs by decreasing the mitochondrial electron transport chain and increasing aerobic glycolysis at all oxygen concentration [53].

Placental syncytiotrophoblasts require members of the ATP-binding cassette (ABC) and solute carrier (SLC) transport families for the transport of nutrients and hormones [54]. Altered expression of a large number of transporters from both these families in TGCs compared to TSCs reflects a change in the functional properties of two cell types. Some gene families, with a direct link to TGC function, were exclusively expressed in TGCs. For example, genes encoding for prolactins (PRL), involved in maintenance of pregnancy, keratins (KRT), involved in cytoskeletal remodeling of trophoblasts, and pregnancy-specific glycoproteins (PSG), involved in immune and metabolic regulation during pregnancy, were all expressed predominantly in TGCs [55–57]. Likewise, an increase in the expression of 15 genes encoding histone proteins in TGCs represents an increased histone demand for a polyploid genome in TGCs. Expression of histone variants at the expense of conventional histones in TGCs has been shown to be responsible for a loosely arranged chromatin structure in TGCs [58]. Instead, we found an increase in the expression of most conventional histone and a decrease in the expression of most histone variants in TGCs. Further studies are needed to reconcile this difference.

TGCs are migratory and invade the uterus during embryo implantation secreting various proteases and cytokines along the way [59, 60]. Defective invasion during implantation of the embryo can result in placental anomalies. For example, in preeclampsia, invasion of the interstitial uterine compartment by the cytotrophoblast is shallow, and spiral artery invasion is incomplete [61]. Anomalies can also arise if the placenta goes beyond the limits due to extensive invasion and invades local structures; these include placenta accreta, placenta increta, and placenta percreta [62]. We observed altered expression of several previously unreported genes involved in cell migration and invasion, which could help develop a deeper understanding of the factors involved in embryo implantation. For example, *Synpo2* which promotes cell migration by focal adhesion assembly and actin bundle formation [63] was expressed at elevated levels in TGCs. Similarly, we discovered that genes encoding 13 different cathepsin proteases, some of which are known to promote trophoblast differentiation and invasion [64], were upregulated in TGCs.

Differentiation into TGCs is associated with the reorganization of the actin cytoskeleton and large focal adhesion formation [65]. We also found extensive rearrangements of the actin cytoskeleton in the form of stress fiber formation in TGCs (Fig. S7). This is supported by the upregulation of several myosin genes and regulators of Rho GTPases in TGCs, two key protein families involved in stress fiber formation. Expression of LIM domain kinase 1 (*Limk1*), involved in the regulation of actin microfilament and control of microtubule dynamics [66], was also upregulated by 3.5-folds. LIMK1 downregulation is associated with preeclampsia [67]; its upregulation in TGCs, therefore, could regulate extensive cytoskeletal rearrangements upon differentiation and thereby may have a role in implantation. Genes encoding tubulin proteins (*Tubb1* and *Tubal3*) and proteins involved in tubulin polymerization were upregulated in TGCs. For example, genes encoding microtubule stabilizing proteins, microtubule-associated protein 1B (*Map1b*) [68], stabilizer of axonemal microtubules 1 (*Saxo1*) [69], tubulin polymerization promoting protein family member 3 (*Tppp3*) [70], and tektin family members (*Tekt2*, *Tekt3*, and *Tekt4*) [71] were all upregulated in TGCs. Uterine TPPP3 expression is reported to play an important role in embryo implantation through the TGF beta pathway [72]. Expression of *Capn6* gene encoding another microtubule-stabilizing protein [73], however, was downregulated in TGCs. Genes for microtubule destabilizing stathmin-like proteins (*Stmn3* and *Stmn4*) were also upregulated in TGCs; increased expression of stathmin is associated with trophoblast migration and differentiation [74]. The upregulation of both microtubule stabilizing and destabilizing proteins indicates the highly dynamic nature of microtubules during and after differentiation and is consistent with TGC function. Inhibition of microtubule dynamics with microtubule depolymerizing drug, colchicine, does inhibit differentiation of trophoblasts [75]. Remodeling of the microtubule and actin cytoskeleton together with the exclusive upregulation of 15 genes from the largest intermediate filament protein family (keratins) indicates a major role cytoskeleton plays in differentiation and function of mouse trophoblasts.

RNA-seq analysis in this study revealed three times more differentially expressed genes associated with the differentiation of mouse trophoblast cells as compared to the fusion of BeWo cells [21, 76]. TGCs are distinct from fused BeWo cells in several ways. For example, unlike fused BeWo cells, TGCs continue to synthesize DNA after differentiation and attain a higher level of polyploidy. They have a modified cell cycle in which cells do not go through the M-phase, which is required for cell division. BeWo cells, on the contrary, do not undergo genome duplication through DNA synthesis [77]. Moreover, TGCs are derived from developmentally normal TSCs while BeWo is a cancer cell line and hence is likely to have an altered gene expression profile. Differential expression from either of these studies may not be a complete representation of molecular events controlling trophoblast differentiation during placenta formation. We did, however, observe evolutionarily conserved expression patterns for 229 upregulated and 176 downregulated genes (not less than 1.5-fold) during differentiation of mouse trophoblasts (at 4 days) and fusion of BeWo cells (at 3 days) (Table S4). For example, *Cdk1* is downregulated both during differentiation of TSCs (− 2.27) and fusion of BeWo cells (− 1.6). The function of *Cdk1* is also conserved as its inhibition induces both, the differentiation in TSCs and the fusion in BeWo cells [15, 20]. Other examples of genes with similar expression pattern in both systems include several upregulated (*Cd68*, *Basp1*, *Cyp11a1*, *Egfr*, *Nostrin*, *Cdkn1a*, *Gata3*) and downregulated (*Plk1*, *Ccnb1*, *Cdk18*, *Gata6*, *Hmgb2*, *Myoz1* and *Bmp4*) genes (Table S4) [21, 76].

Alternative splicing produces functionally distinct proteins from the same gene by generating transcript variants and is shown to be associated with several biological processes including stem cell differentiation [78, 79] and fusion of BeWo cell [76]. The presence or function of alternative splicing in the differentiation of mouse TSCs, however, has not been investigated. This study reveals TSC- or TGC-specific expression of exons in 31 genes that are involved in cell growth, invasion, migration, apoptosis, and differentiation. Five different exons of slow skeletal muscle troponin T (*Tnnt1*) gene, for example, were explicitly expressed in TSCs. *Tnnt1* is involved in slow skeletal muscle contraction and is highly expressed in human induced pluripotent stem cells (hiPSCs) and immortalized retinal pigment epithelial (RPE) cell lines. The increased expression serves as a marker for immortalization in RPE cells; the TSC-specific expression of full-length isoform could, therefore, also represent the “immortal” identity of these cells. Tnnt1 overexpression also enhances migration and actin polymerization in RPE cells. A shift in expression from a full-length Tnnt1 protein (in TSCs) to a shorter isoform in TGCs may have a similar role in migration and actin dynamics in TGCs [80, 81]. Other genes with differentially expressed exons are known to be involved in several biological processes including cell growth, invasion, migration, apoptosis, and differentiation [82, 83]. Cell type-specific expression of the identified exons may be important in maintaining stemness or inducing differentiation of trophoblast stem cells. Overexpression or ablation of these exons in TSCs or TGCs can be used to determine any specific role they may play in differentiation of TSCs into TGCs.

RNA-seq data also revealed 19 previously unannotated exons in 12 different genes. One of the genes in which two novel exons are identified is *Map2k2*. Map2k2 (mitogen-activated protein kinase kinase 2) has been reported to play a role in the formation of multinucleated TGCs during placentation [84]. Expression of putative novel exons could generate novel splice variants encoding proteins with TSC- or TGC-specific functions. The identification of novel exons, not present in the current annotations of mouse genes, highlights the weakness of existing mouse gene catalogs and thereby will help in filling the gap in the mouse transcriptome.

## Conclusions

In conclusion, our data points to transcriptional diversity and differential transcriptome in mouse TSCs and TGCs that was not known previously. Differential expression of some of these genes indicates a shift in the functional properties of TGCs as they differentiate from TSCs. Functional validation of these genes will not only enhance our understanding of mammalian development but could also lead to finding ways to treat placental abnormalities and diseases which often lead to premature childbirth.

## Supplementary information

**Additional file 1.**

**Additional file 2.**

**Additional file 3.**

**Additional file 4.**

**Additional file 5.**

**Additional file 6.**

**Additional file 7.**

**Additional file 8.**

**Additional file 9: Figure S1.** Venn diagram showing the number of differentially regulated genes identified as significant by DESEQ2 and EdgeR in TGCs compared to TSCs. The overlap shows the number of differentially regulated genes identified both by DESEQ2 and EdgeR.

**Additional file 10: Figure S2.** Gene Ontology enrichment analysis of differentially expressed genes in TGCs in the Biological Process **a**, Molecular function **b** and Cellular component **c** categories. The selected 10 GO terms in each category were over-represented by > 2-fold enrichment value, with FDR values < 0.05. Fold enrichment values are given with each GO term on X-axis.

**Additional file 11: Figure S3.** Differentiation phenotype induced by the Aurora inhibitors in TSCs. TSCs were treated with 1 μM concentration of Aurora inhibitors (identified in the primary chemical genetic screen; Table S3 and unpublished data) in 96-well plates for 72 h. Cells were fixed with paraformaldehyde and stained with phalloidin (*green*) and DAPI (*blue*). Images were taken at 60x magnification.

**Additional file 12: Figure S4.** Experimental validation of differentially expressed exons in 9 selected genes. **a** IGV snapshots of the selected differentially expressed exons. Read coverage for TG-specific exons in *Tmem40*, *P2ry2* and *Ptprk* genes and TS-specific exons in *Garnl3*, *Lrrfip*, *Lmo7*, *Dnmbp*, *Kalrn* and *Asic1* genes are shown in all three replicates. Reference gene track is shown at the bottom (*gray line*) with bars representing the corresponding exons and arrowhead (*black*) showing the orientation of the gene. **b** The primer pair design strategy for the amplification of differentially expressed exons. Primer pairs for the amplification of differentially expressed exons in *Tmem40*, *Ptprk*, *Lrrfip*, *Asic1*, *Garnl3* and *Lmo7* were designed in different exons while in *Dnmbp*, *P2ry2 and Kalrn*, the primer pairs were designed only in differentially expressed exons. Rectangles represent differentially expressed (*black*) and non-differentially expressed (*dark gray*) exons while arrows (*black*) represent forward (*top*) and reverse (*bottom*) primers. The two exons are separated by the intronic region (black line). **c** mRNA expression analysis of differentially expressed exons in TSCs and TGCs by quantitative real-time PCR. Actin was employed as an internal control for all genes. Error bars represent SEM of 3 independent biological replicates.

**Additional file 13: Figure S5.** Experimental validation of novel exon identified in 8 selected genes. **a** IGV snapshot of the identified novel exons showing read coverage in all 3 replicates of TSCs (*red*) and TGCs (*blue*). Reference gene track is shown at the bottom (*blue*) with bars representing the corresponding exons and arrowhead (*black*) showing the orientation of the gene. Novel exons are enclosed by red boxes. **b** The primer pair design strategy for the amplification of novel exons. Primer pairs for the amplification of novel exons in *Asprv1*, *Zbtb7c*, *Eps8l1*, *Snx29* and *Gramd1c* were designed in different exons while in *Sil1*, *Ai506816* and *Map2k2*, the primer pairs were designed only in novel identified exons. Rectangles represent novel (*red*) and known (*blue*) exons while arrows (*black*) represent forward (*top*) and reverse (*bottom*) primers. The two exons are separated from each other by a line (*black*) representing intronic region. **c** PCR amplification of novel exons from TSCs and TGCs. Agarose gel image of the PCR amplified products of novel exon in 9 genes. Amplification of actin from RT+ and RT- (with and without reverse transcriptase) represents positive and negative controls, respectively.

**Additional file 14: Figure S6.** Novel gene models predicted by AUGUSTUS. Gene models were built for all 14 different genes, showing existing exons (*dark blue*) and novel exons (*red*).

**Additional file 15: Figure S7.** Cytoskeleton staining in TSCs and TGCs with phalloidin, which stains actin (*green*) and alpha-tubulin (*red*). Nuclei were stained with DAPI (*blue*).

**Additional file 16: Table S1.** After sequencing, the raw reads were filtered. Data filtering included removing adaptor sequences, contamination and low-quality reads from raw reads. The table shows statistical results after data treatment. **Table S2.** The given log fold change values for each gene were detected by EdgeR. Positive values represent fold upregulation while negative values represent fold down-regulation. References for the reported differential expression is also shown. **Table S3.** Inhibition of Aurora kinases induces differentiation phenotype (increased nuclear size) in TSCs. Fold increase in nuclear size of TSCs after treatment with 1 μM concentration of various Aurora kinase inhibitors (A-specific, Aurora B-specific and pan-Aurora inhibitors) for 72 h in the primary and secondary screens. Relative nuclear size was calculated from the DAPI stained cells in 96-well plates (3 images per well). **Table S4.** Common differentially expressed genes (upregulated and downregulated) identified in 4 days differentiated TGCs RNA-seq data and 3 days fused BeWo RNA-seq data. **Table S5.** Primers used for experimental validation of differentially expressed genes, differentially expressed exons and novel identified exons [7, 15, 16, 33, 85–93].

## Data Availability

The RNA-seq data files from this study have been submitted to the NCBI Sequence Read Archive (SRA; http://www.ncbi.nlm.nih.gov/sra) under accession number SRP274148.

## Abbreviations

bp: Base pair
BSA: Bovine serum albumin
cDNA: Complementary deoxyribonucleic acid
DAPI: (4′,6-diamidino-2-phenylindole)
dNTP: Deoxynucleotide triphosphate
EDTA: Ethylene-diamine-tetra-acetic acid
ERR1: Estrogen-related receptor alpha
ES-FBS: Embryonic stem cell fetal bovine serum
FACS: Fluorescence activated cell sorting
FGF4: Fibroblast growth factor 4
FGFR2: Fibroblast growth factor receptor 2
MgCl_2_: Magnesium chloride
mRNA: Messenger ribonucleic acid
NaCl: Sodium chloride
NaF: Sodium fluoride
PBS: Phosphate buffer saline
PCR: Polymerase chain reaction
PL-1: Placental lactogen-1
PMEF: Primary mouse embryonic fibroblast
RNA: Ribonucleic acid
RT: Reverse transcriptase
Taq: *Thermus aquaticus*
TGCs: Trophoblast giant cells
TPBPA: Trophoblast-specific protein alpha
TSCs: Trophoblast stem cells
