# Supplementary figures and images for: Transcriptomic analysis reveals differential gene expression, alternative splicing, and novel exons during mouse trophoblast stem cell differentiation
Source: Stem Cell Res Ther. 2020 Aug 6;11:342. doi: 10.1186/s13287-020-01848-8 (PMC7409654; doi:10.1186/s13287-020-01848-8)

DESEQ2 significant genes

EdgeR significant genes

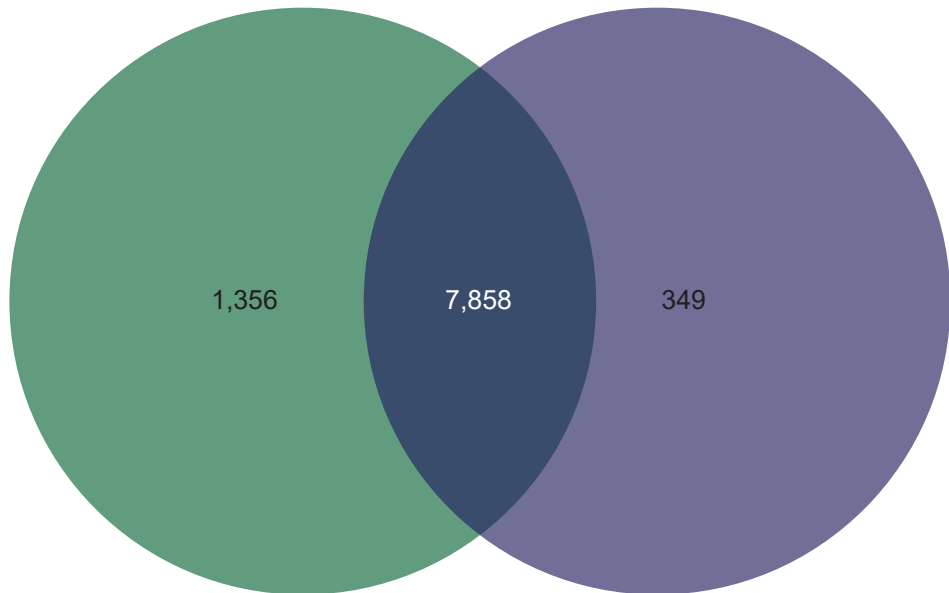

Supplement: Supplementary file 9 — Additional file 9: Figure S1. Venn diagram showing the number of differentially regulated genes identified as significant by DESEQ2 and EdgeR in TGCs compared to TSCs. The overlap shows the number of differentially regulated genes identified both by DESEQ2 and EdgeR. [file 13287_2020_1848_MOESM9_ESM.pdf]

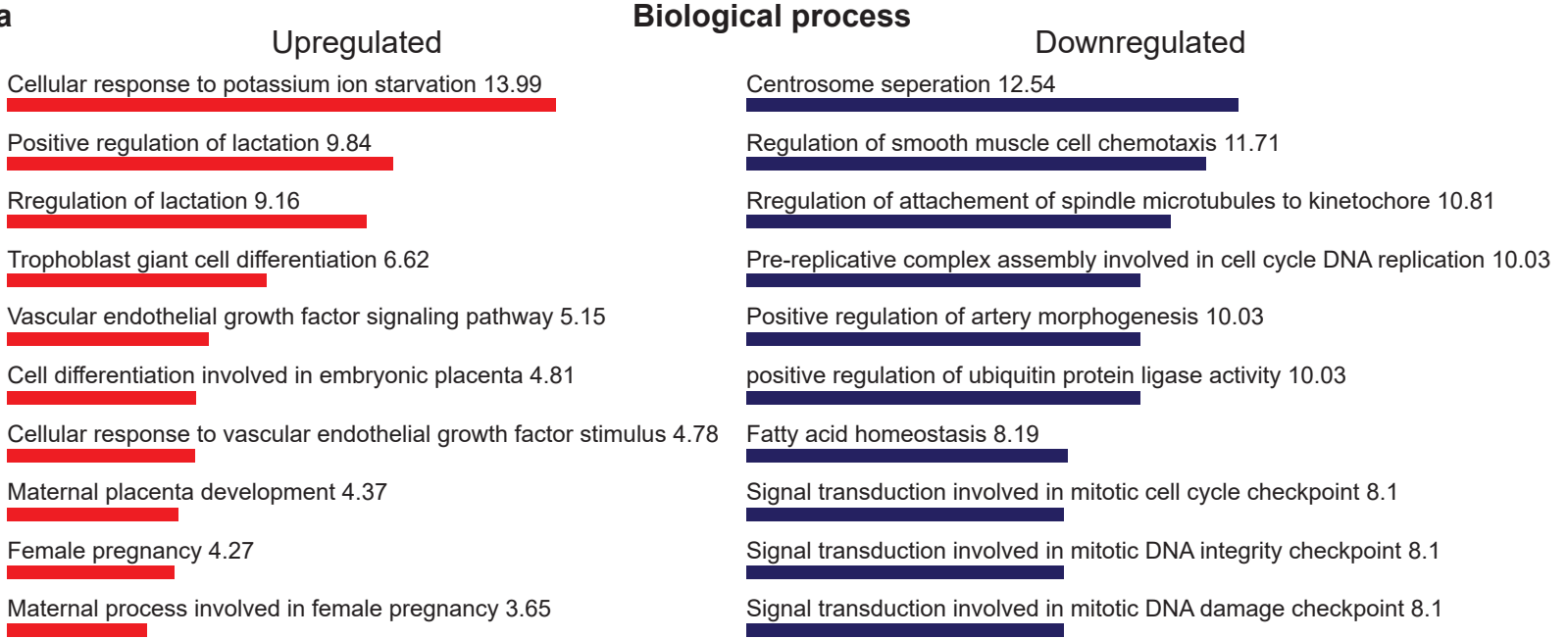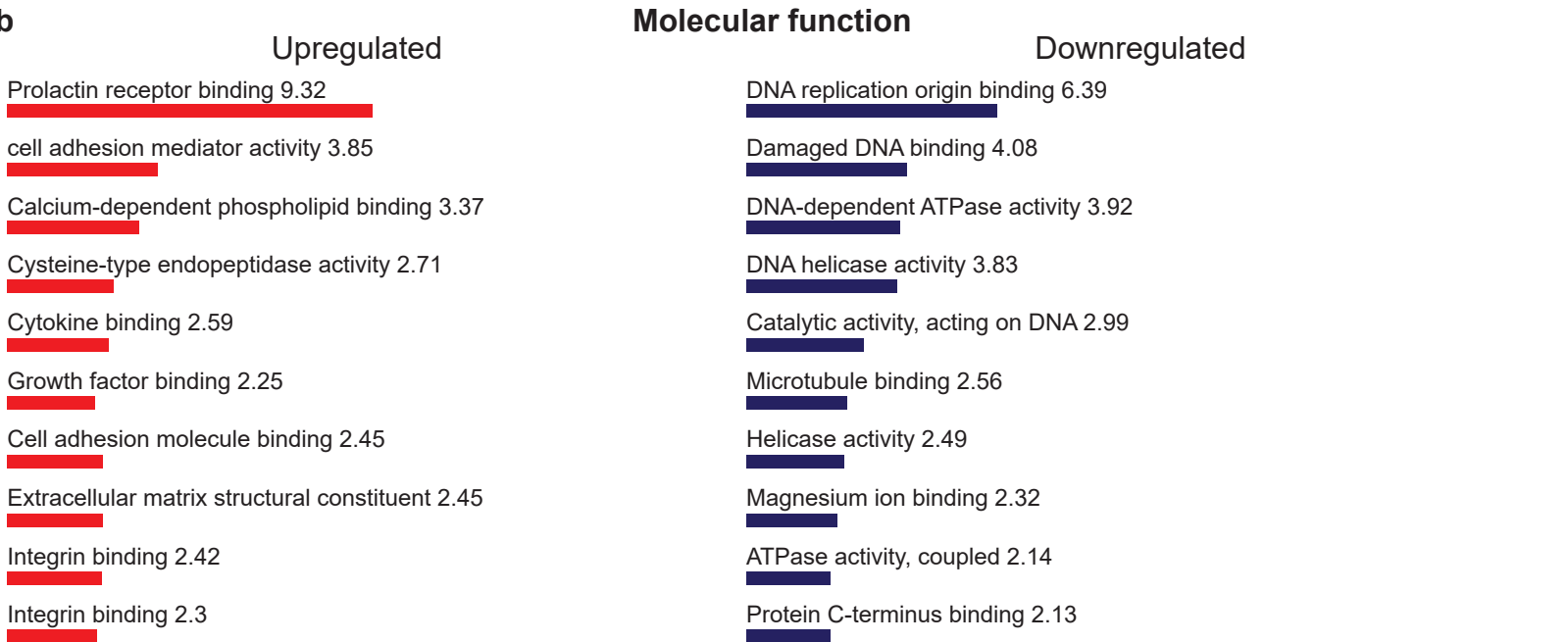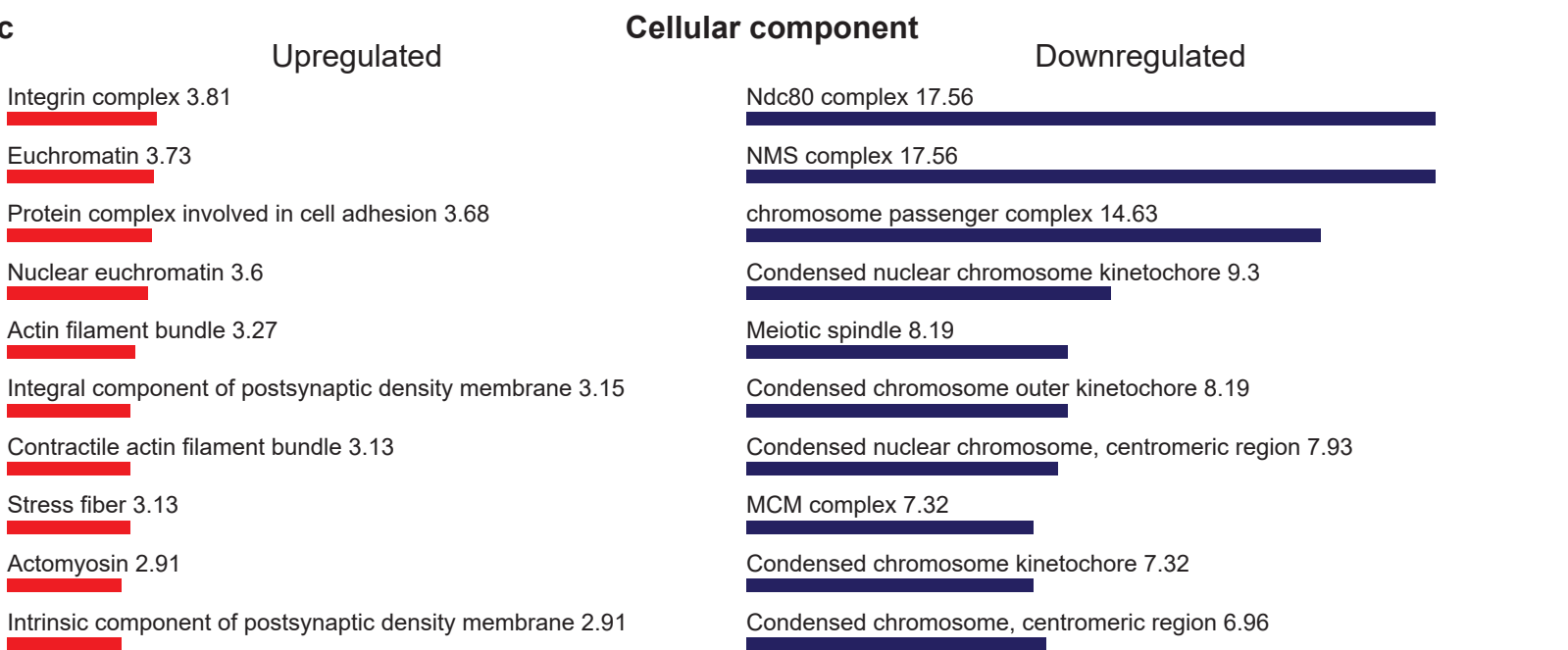

Supplement: Supplementary file 10 — Additional file 10: Figure S2. Gene Ontology enrichment analysis of differentially expressed genes in TGCs in the Biological Process a, Molecular function b and Cellular component c categories. The selected 10 GO terms in each category were over-represented by > 2-fold enrichment value, with FDR values < 0.05. Fold enrichment values are given with each GO term on X-axis. [file 13287_2020_1848_MOESM10_ESM.pdf]

TSCs

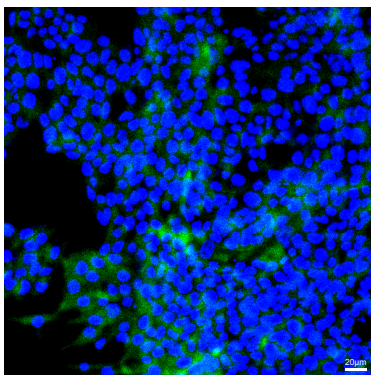

TGCs

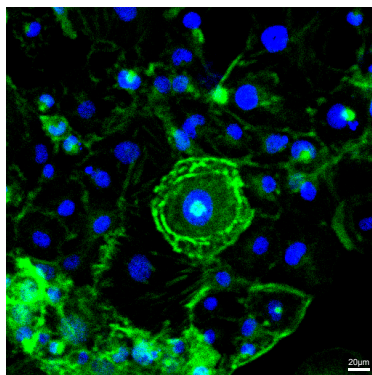

MLN8237

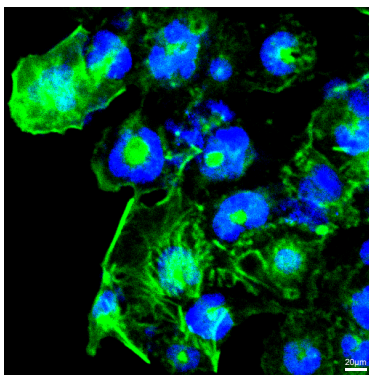

MLN8054

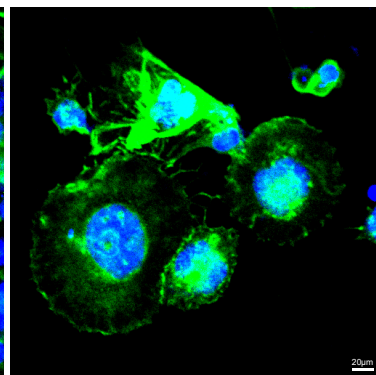

MK-8745

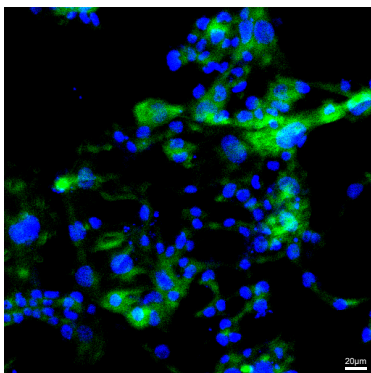

AZD1152

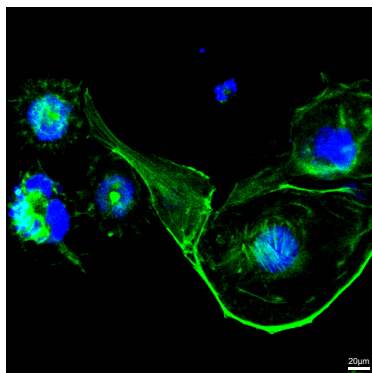

GSK1070916

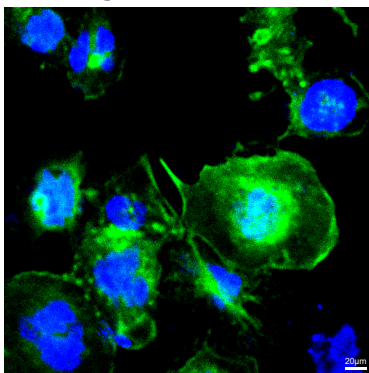

VX-680

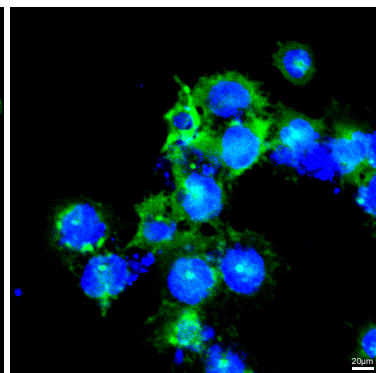

PHA739358

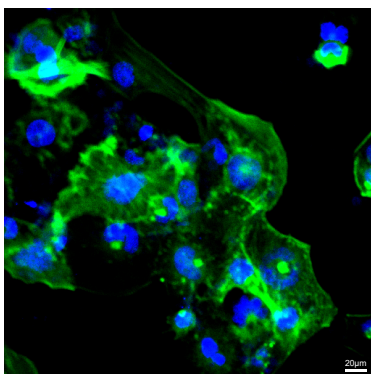

CCT137690

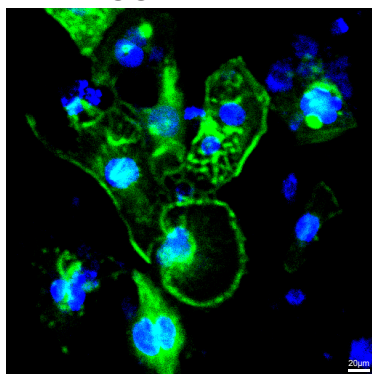

SNS-314

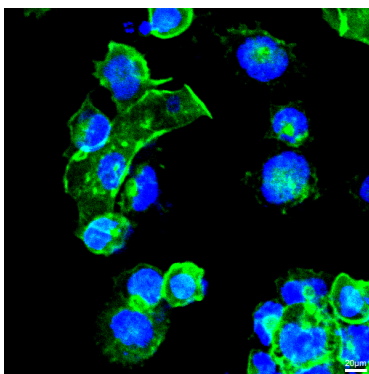

AMG-900

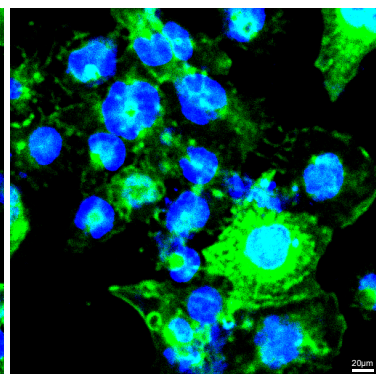

CYC116

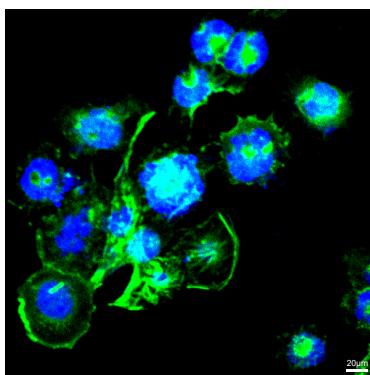

Supplement: Supplementary file 11 — Additional file 11: Figure S3. Differentiation phenotype induced by the Aurora inhibitors in TSCs. TSCs were treated with 1 μM concentration of Aurora inhibitors (identified in the primary chemical genetic screen; Table S3 and unpublished data) in 96-well plates for 72 h. Cells were fixed with paraformaldehyde and stained with phalloidin (green) and DAPI (blue). Images were taken at 60x magnification. [file 13287_2020_1848_MOESM11_ESM.pdf]

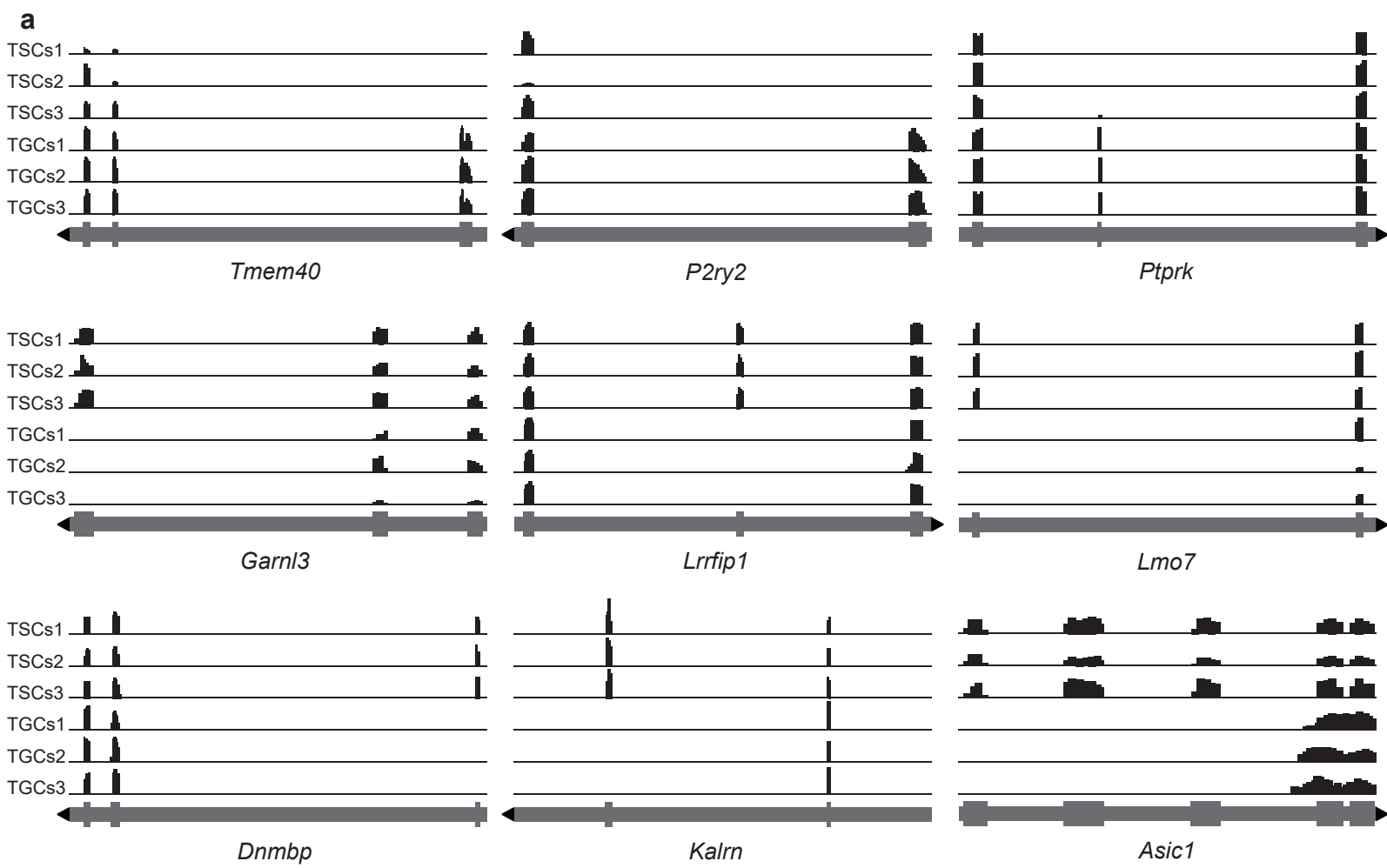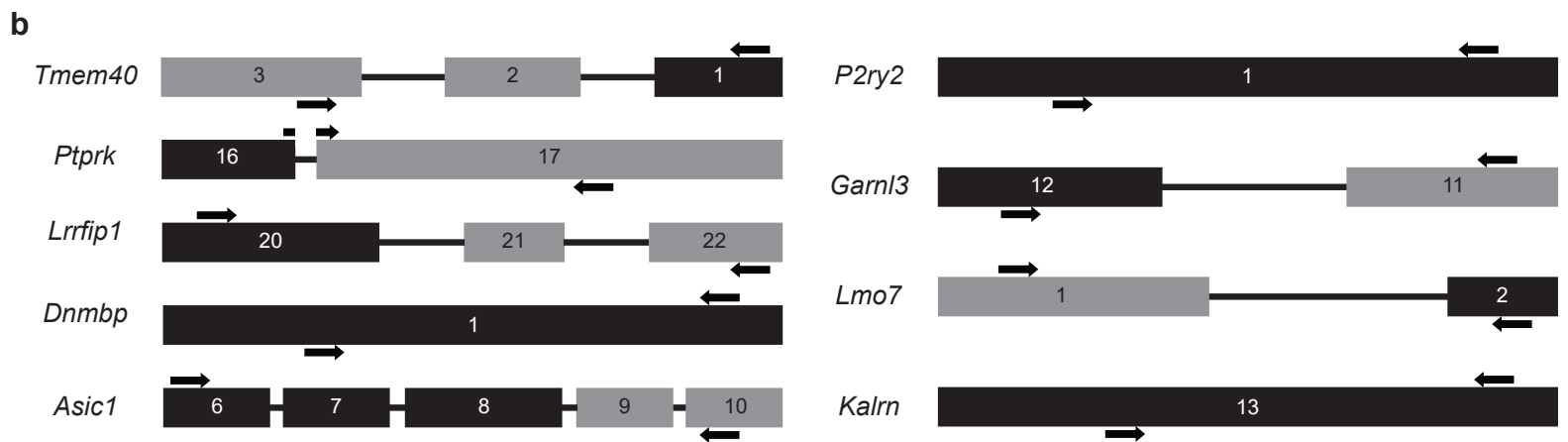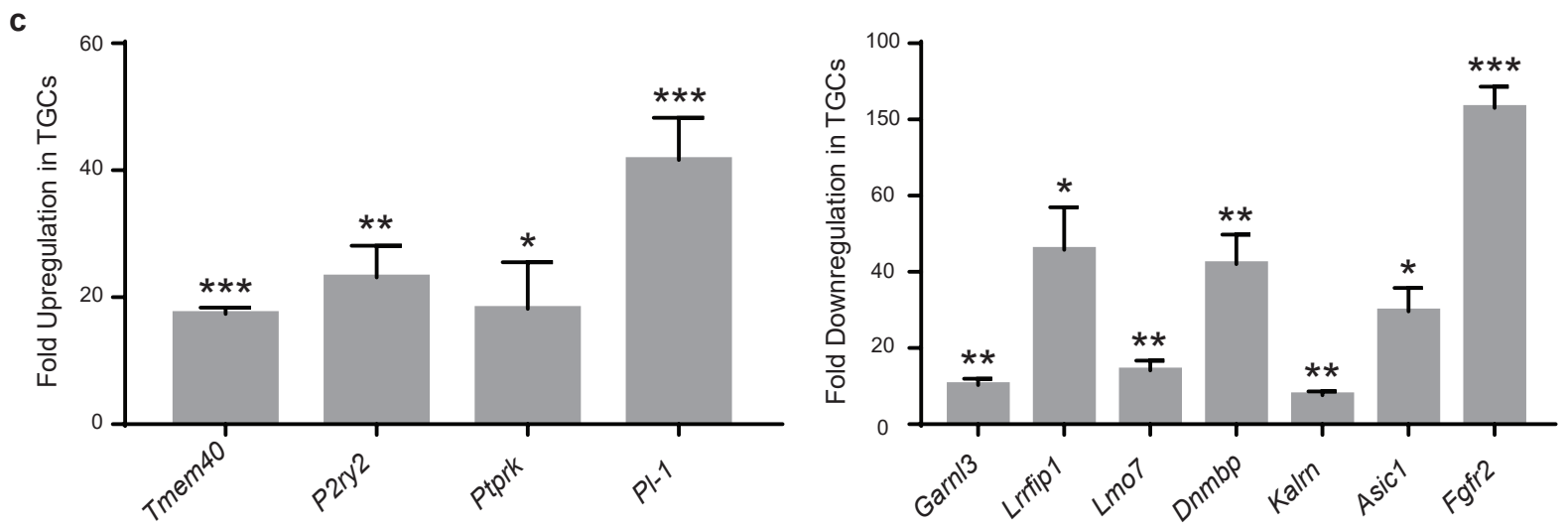

Supplement: Supplementary file 12 — Additional file 12: Figure S4. Experimental validation of differentially expressed exons in 9 selected genes. a IGV snapshots of the selected differentially expressed exons. Read coverage for TG-specific exons in Tmem40, P2ry2 and Ptprk genes and TS-specific exons in Garnl3, Lrrfip, Lmo7, Dnmbp, Kalrn and Asic1 genes are shown in all three replicates. Reference gene track is shown at the bottom (gray line) with bars representing the corresponding exons and arrowhead (black) showing the orientation of the gene. b The primer pair design strategy for the amplification of differentially expressed exons. Primer pairs for the amplification of differentially expressed exons in Tmem40, Ptprk, Lrrfip, Asic1, Garnl3 and Lmo7 were designed in different exons while in Dnmbp, P2ry2 and Kalrn, the primer pairs were designed only in differentially expressed exons. Rectangles represent differentially expressed (black) and non-differentially expressed (dark gray) exons while arrows (black) represent forward (top) and reverse (bottom) primers. The two exons are separated by the intronic region (black line). c mRNA expression analysis of differentially expressed exons in TSCs and TGCs by quantitative real-time PCR. Actin was employed as an internal control for all genes. Error bars represent SEM of 3 independent biological replicates. [file 13287_2020_1848_MOESM12_ESM.pdf]

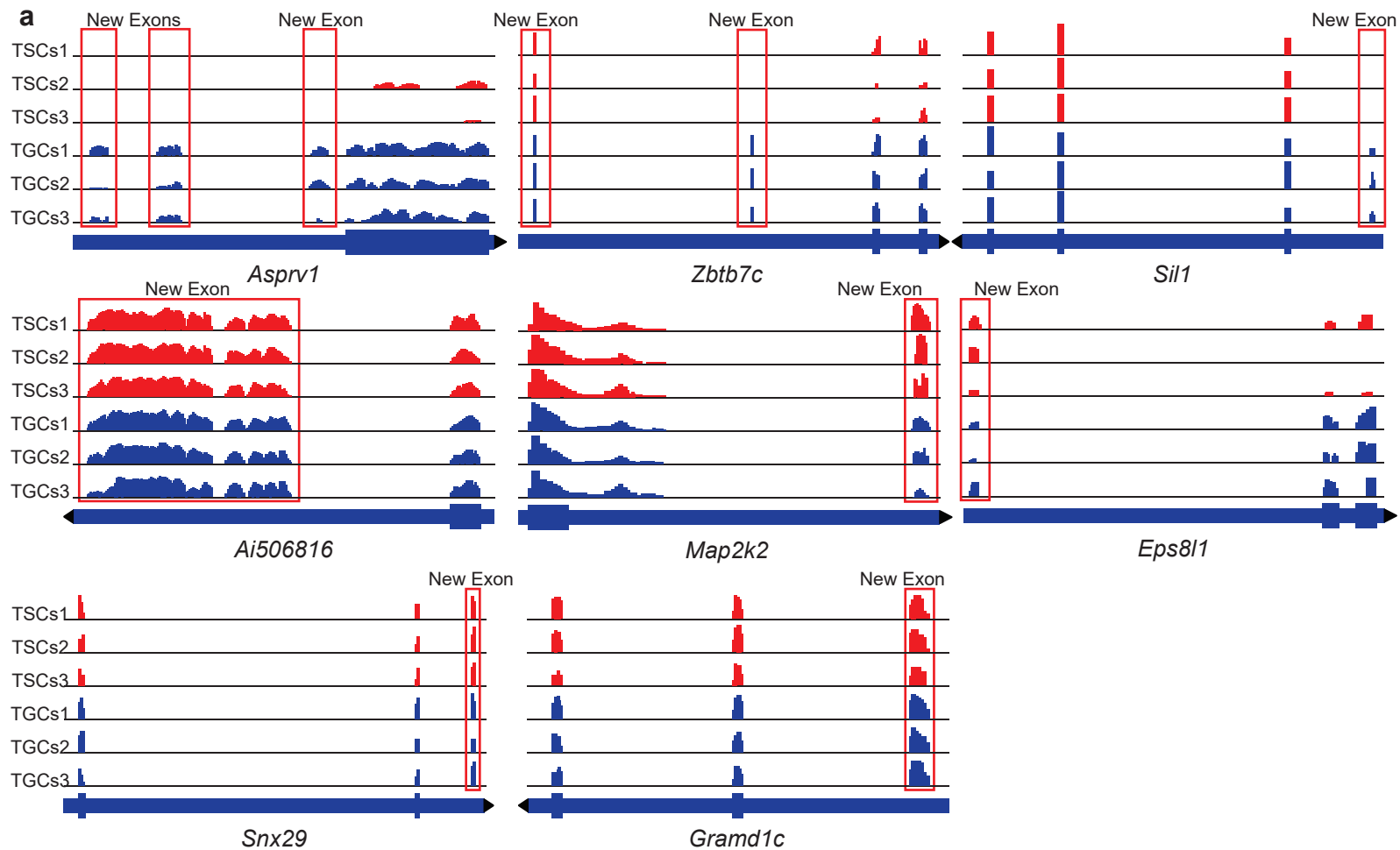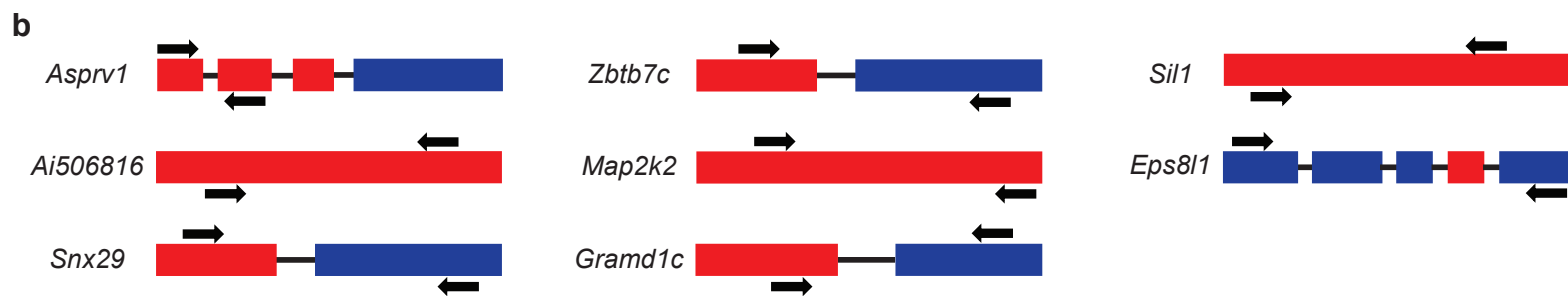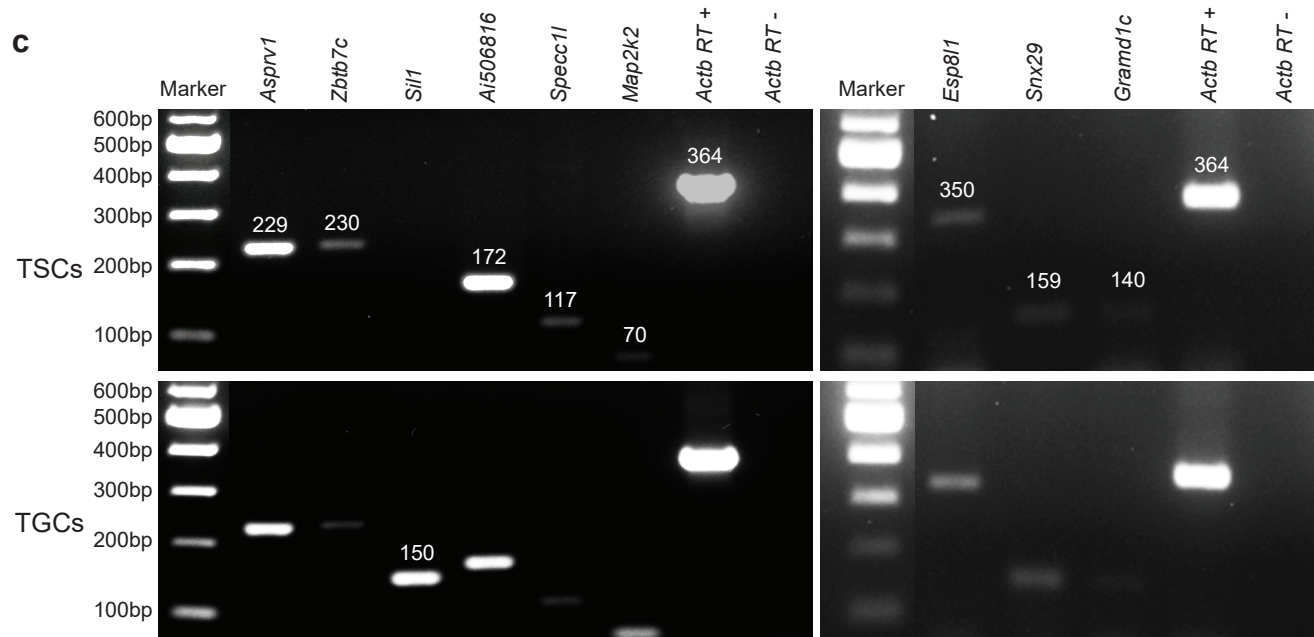

Supplement: Supplementary file 13 — Additional file 13: Figure S5. Experimental validation of novel exon identified in 8 selected genes. a IGV snapshot of the identified novel exons showing read coverage in all 3 replicates of TSCs (red) and TGCs (blue). Reference gene track is shown at the bottom (blue) with bars representing the corresponding exons and arrowhead (black) showing the orientation of the gene. Novel exons are enclosed by red boxes. b The primer pair design strategy for the amplification of novel exons. Primer pairs for the amplification of novel exons in Asprv1, Zbtb7c, Eps8l1, Snx29 and Gramd1c were designed in different exons while in Sil1, Ai506816 and Map2k2, the primer pairs were designed only in novel identified exons. Rectangles represent novel (red) and known (blue) exons while arrows (black) represent forward (top) and reverse (bottom) primers. The two exons are separated from each other by a line (black) representing intronic region. c PCR amplification of novel exons from TSCs and TGCs. Agarose gel image of the PCR amplified products of novel exon in 9 genes. Amplification of actin from RT+ and RT- (with and without reverse transcriptase) represents positive and negative controls, respectively. [file 13287_2020_1848_MOESM13_ESM.pdf]

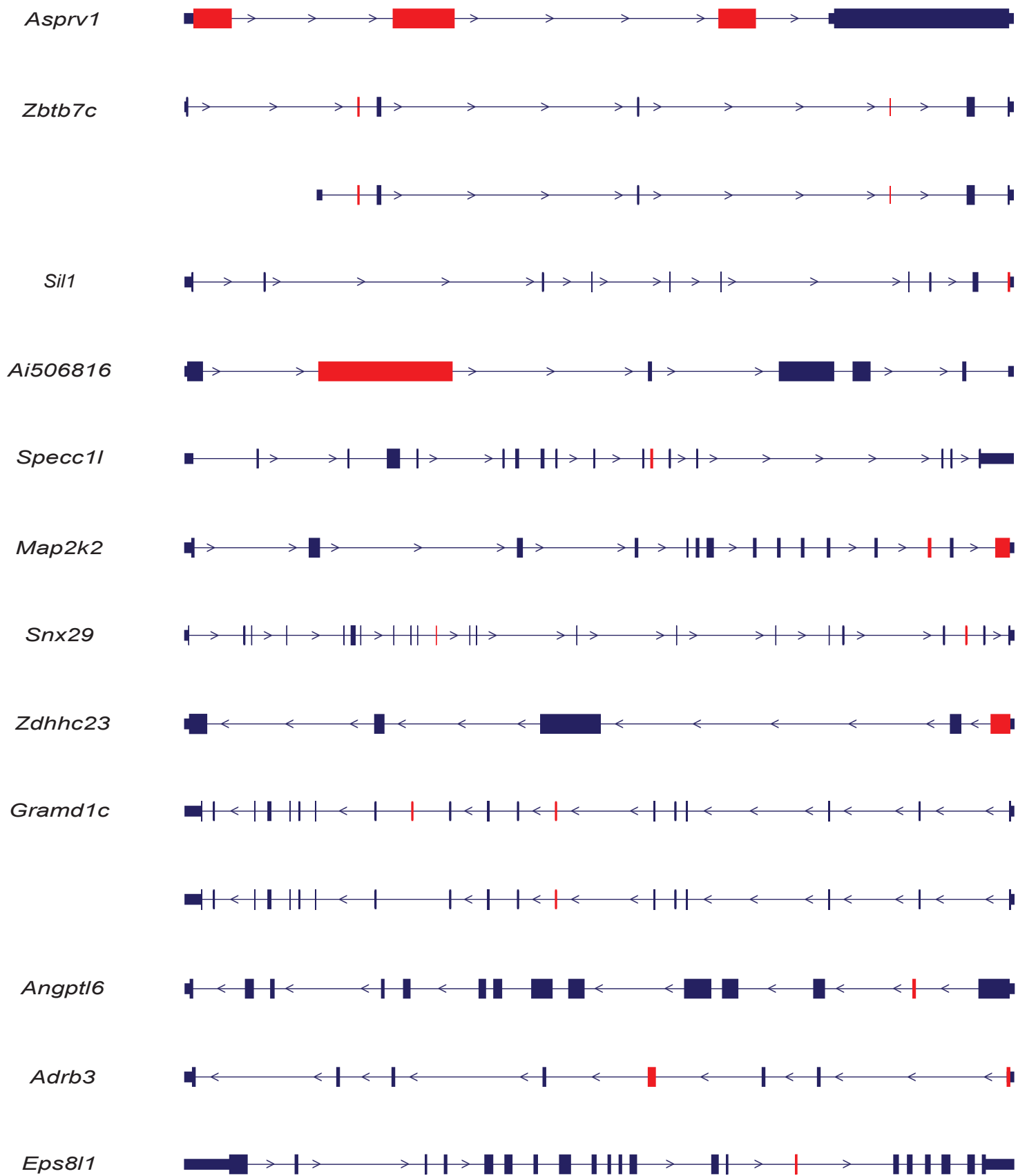

Supplement: Supplementary file 14 — Additional file 14: Figure S6. Novel gene models predicted by AUGUSTUS. Gene models were built for all 14 different genes, showing existing exons (dark blue) and novel exons (red). [file 13287_2020_1848_MOESM14_ESM.pdf]

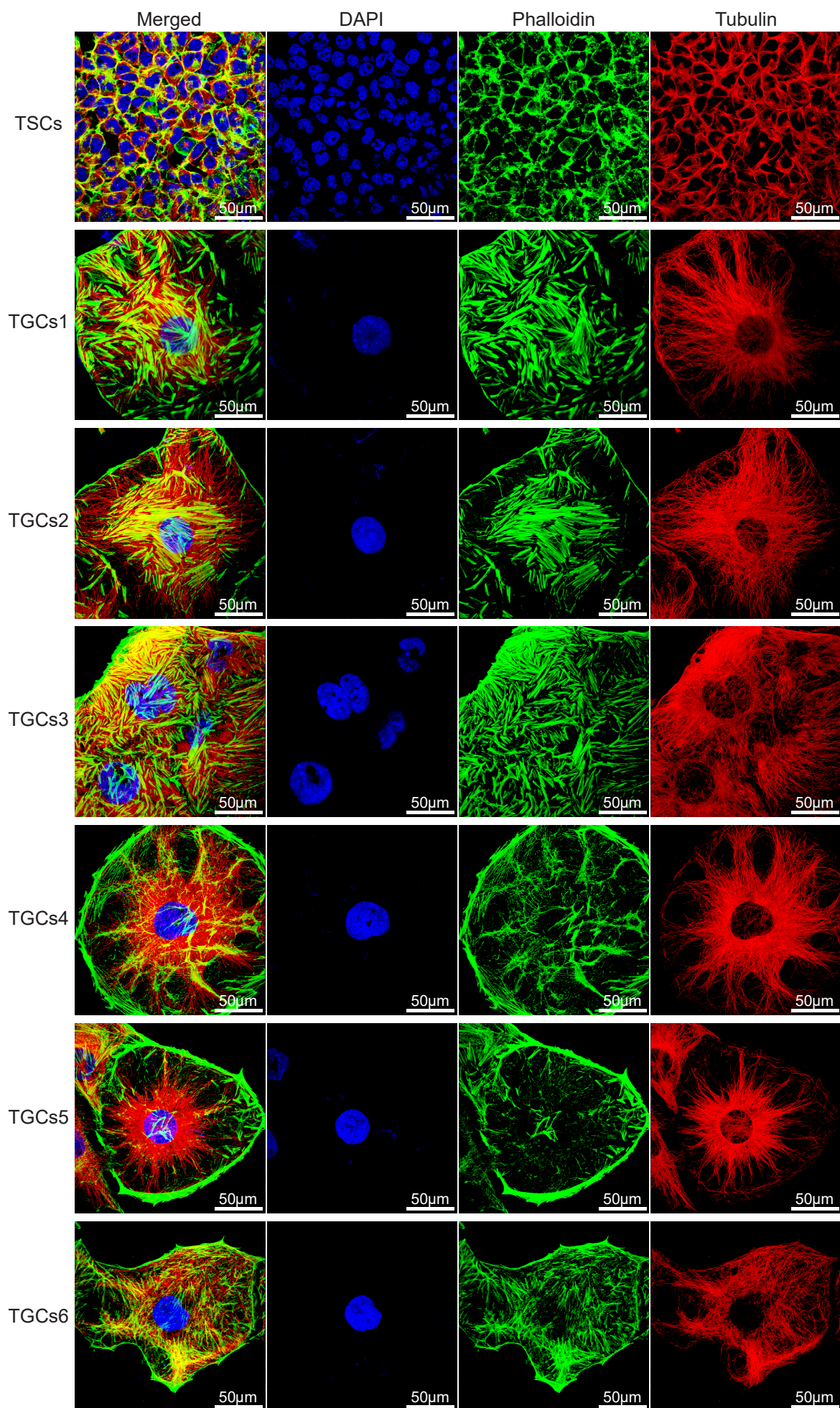

Supplement: Supplementary file 15 — Additional file 15: Figure S7. Cytoskeleton staining in TSCs and TGCs with phalloidin, which stains actin (green) and alpha-tubulin (red). Nuclei were stained with DAPI (blue). [file 13287_2020_1848_MOESM15_ESM.pdf]
