# Supplementary material for: Transcriptomic analysis reveals differential gene expression, alternative splicing, and novel exons during mouse trophoblast stem cell differentiation
Source: Stem Cell Res Ther. 2020 Aug 6;11:342. doi: 10.1186/s13287-020-01848-8 (PMC7409654; doi:10.1186/s13287-020-01848-8)
Supplement: Supplementary file 16 — Additional file 16: Table S1. After sequencing, the raw reads were filtered. Data filtering included removing adaptor sequences, contamination and low-quality reads from raw reads. The table shows statistical results after data treatment. Table S2. The given log fold change values for each gene were detected by EdgeR. Positive values represent fold upregulation while negative values represent fold down-regulation. References for the reported differential expression is also shown. Table S3. Inhibition of Aurora kinases induces differentiation phenotype (increased nuclear size) in TSCs. Fold increase in nuclear size of TSCs after treatment with 1 μM concentration of various Aurora kinase inhibitors (A-specific, Aurora B-specific and pan-Aurora inhibitors) for 72 h in the primary and secondary screens. Relative nuclear size was calculated from the DAPI stained cells in 96-well plates (3 images per well). Table S4. Common differentially expressed genes (upregulated and downregulated) identified in 4 days differentiated TGCs RNA-seq data and 3 days fused BeWo RNA-seq data. Table S5. Primers used for experimental validation of differentially expressed genes, differentially expressed exons and novel identified exons [7, 15, 16, 33, 85–93]. [file 13287_2020_1848_MOESM16_ESM.docx]

**Supplementary Tables**

| **Sample** | **Clean reads** | **Clean bases** | **Read length (bp)** | **Q20(%)** | **GC(%)** |
| --- | --- | --- | --- | --- | --- |
| TSCs1 | 40,169,360 | 4,016,936,000 | 100 | 96.78%; 95.66% | 51.39% |
| TGCs1 | 44,167,172 | 4,416,717,200 | 100 | 96.55%; 95.76% | 51.44% |
| TSCs2 | 40,390,214 | 4,039,021,400 | 100 | 96.85%; 95.22% | 50.99% |
| TGCs2 | 40,297,514 | 4,029,751,400 | 100 | 97.23%; 96.36% | 51.55% |
| TSCs3 | 40,144,704 | 4,014,470,400 | 100 | 97.80%; 95.70% | 51.20% |
| TGCs3 | 40,465,592 | 4,046,559,200 | 100 | 97.85%; 96.19% | 51.59% |

**Table S1.** After sequencing, the raw reads were filtered. Data filtering included removing adaptor sequences, contamination and low-quality reads from raw reads. The table shows statistical results after data treatment.

| **Gene** | **Description** | **Regulation** | **Log fold change** | **References** |
| --- | --- | --- | --- | --- |
| *Ctsq* | Cathepsin Q | Upregulated | 9.756412 | (7) |
| *Pl1* | Prolactin family 3, subfamily d, member 1 | Upregulated | 9.734492 | (33) |
| *Pl2* | Prolactin family 3, subfamily b, member 1 | Upregulated | 7.558776 | (7) |
| *Tpbpa* | Trophoblast specific protein alpha | Upregulated | 6.593576 | (7) |
| *Plf* | Prolactin family 2, subfamily c, member 2 | Upregulated | 6.122495 | (7) |
| *Nostrin* | Nitric oxide synthase trafficker | Upregulated | 3.780227 | (85) |
| *P21* | Cyclin-dependent kinase inhibitor 1A | Upregulated | 3.310303 | (86) |
| *Tfap2a* | Transcription factor AP-2, alpha | Upregulated | 3.191446 | (87) |
| *P57* | Cyclin-dependent kinase inhibitor 1C | Upregulated | 2.736346 | (86) |
| *Bcor* | BCL6 interacting corepressor | Upregulated | 1.58097 | (88) |
| *Akt1* | Thymoma viral proto-oncogene 1 | Upregulated | 1.335076 | (86) |
| *Tfap2c* | Transcription factor AP-2, gamma | Upregulated | 0.994363 | (87) |
| *Ccne1* | Cyclin E1 | Downregulated | -3.64095 | (15) |
| *Gmnn* | Geminin | Downregulated | -2.70576 | (89) |
| *Eomes* | Eomesodermin | Downregulated | -1.66111 | (90) |
| *Esrrb* | Estrogen related receptor, beta | Downregulated | -1.29376 | (91) |
| *Cdk1* | Cyclin-dependent kinase 1 | Downregulated | -1.41042 | (15) |
| *Elf5* | E74-like factor 5 | Downregulated | -1.2556 | (33) |
| *Cdx2* | Caudal type homeobox 2 | Downregulated | -1.25521 | (92) |
| *Chk1* | Checkpoint kinase 1 | Downregulated | -0.8192 | (16) |
| *Sox21* | SRY (sex determining region Y)-box 21 | Downregulated | -0.43797 | (93) |

**Table S2.** The given log fold change values for each gene were detected by EdgeR. Positive values represent fold upregulation while negative values represent fold down-regulation. References for the reported differential expression is also shown.

| **Inhibitors name** | **Target protein** | **Fold increase in nuclear size** | |
| --- | --- | --- | --- |
|  |  | **Primary Screen** | **Secondary Screen** |
| TSC media | - | 1 | 1 |
| TGC media | - | 1.43 | 1.61 |
| MLN8237 | Aurora A | 3.62 | 3.57 |
| MLN8054 | Aurora A | 3.36 | 3.68 |
| MK-8745 | Aurora A | 1.32 | 1.43 |
| AZD1152 | Aurora B | 2.95 | 3.68 |
| GSK1070916 | Aurora B | 3.38 | 4.15 |
| VX-680 | Aurora A, B and C | 3.34 | 3.97 |
| PHA739358 | Aurora A, B and C | 1.55 | 1.76 |
| CCT137690 | Aurora A, B and C | 2.54 | 2.52 |
| SNS-314 | Aurora A, B and C | 3.31 | 3.59 |
| AMG-900 | Aurora A, B and C | 3.46 | 3.74 |
| CYC116 | Aurora A, B and C | 3.27 | 3.61 |

**Table S3.** Inhibition of Aurora kinases induces differentiation phenotype (increased nuclear size) in TSCs. Fold increase in nuclear size of TSCs after treatment with 1 µM concentration of various Aurora kinase inhibitors (A-specific, Aurora B-specific and pan-Aurora inhibitors) for 72 hours in the primary and secondary screens. Relative nuclear size was calculated from the DAPI stained cells in 96-well plates (3 images per well).

| **Common genes** | | | | | |
| --- | --- | --- | --- | --- | --- |
| **Upregulated** | | | **Downregulated** | | |
| *Cyp11a1* | *Fstl3* | *Tgm2* | *Tpst1* | *Ide* | *Parp3* |
| *Atf3* | [*Herpud1*](http://www.informatics.jax.org/marker/MGI:1927406) | *Eps8l1* | *Sema5a* | *Pou6f1* | *Iqgap3* |
| *Egfr* | *Gdf15* | *Basp1* | *Ybx2* | *Rassf2* | *Zic5* |
| *Sdc1* | *Bhlhe40* | *Sema7a* | *Hip1* | *Myoz1* | *Spred1* |
| *Cpeb4* | *Bhlha15* | *Fn1* | *Prps1* | *Wnt7b* | *Wasf1* |
| *Clec1a* | *Mtus1* | *Tnfaip2* | *Mpp2* | *Cdk18* | *Coro1a* |
| *Mvp* | *Csrp1* | *Fdx1* | *Fam171a2* | *Ccdc88c* | *Rbpms2* |
| *Cdkn1a* | *Nostrin* | *Arhgdib* | *Thpo* | *Smo* | *Psmb9* |
| *Slc6a19* | *Gab2* | *Col6a3* | *Lin28a* | *Kif21b* | *Epcam* |
| *Micall2* | *Stra6* | *Sqstm1* | *Nme4* | *Igsf9* | *Bcl7a* |
| *Tfap2a* | *St3gal4* | *Stom* | *Bmf* | *Bmp4* | *Pls1* |
| *Tgfbr3* | *Lactb* | *Alad* | *Dnmt3l* | *Ogdhl* | *Elovl2* |
| *Slco4a1* | *Pwwp2b* | *Csgalnact1* | *Dlc1* | *Hoxc10* | *Gabbr1* |
| *Cntnap2* | *Slc7a1* | *Arrdc3* | *Mnd1* | *Ninl* | *Gas6* |
| *Atp1a4* | *Ell* | *Plekha6* | *Wnt6* | *Bcat2* | *Gdpd1* |
| *Cdh5* | *Gata3* | *Cd68* | *Csrp2* | *Pif1* | *Casp3* |
| *Maml3* | *Rhob* | *Ppp1r13b* | *Neil3* | *Lmnb1* | *Mybl2* |
| *Fth1* | *Cst6* | *Trpv2* | *Mad2l1* | *Fkbp3* | *Ckap2* |
| *Slc38a3* | *Lgals9* | *Scarb1* | *Pecr* | *Gabrb3* | *Tst* |
| *Oaf* | *Lepr* | *Fgf18* | *Klf15* | *Triml1* | *Aldh5a1* |
| *Nr4a3* | *Dusp8* | *Tnfrsf1b* | *Cdkn3* | *Tm7sf2* | *Dnmt3b* |
| *Greb1* | *Hopx* | *Tmem40* | *Tead2* | *Depdc1a* | *Mxra7* |
| *Pde4d* | *Cyp2c55* | *Sema6a* | *Ccdc34* | *Mfsd3* | *Gldc* |
| *Socs3* | *Psg18* | *Gal3st1* | *Gata6* | *Zic2* | *Plk1* |
| *Slc6a4* | *Anxa1* | *Sectm1b* | *Sh3bgrl* | *Aspm* | *Hmgb2* |
| *Gjb2* | *Nrp1* | *Zfp703* | *Bub1b* | *Bex2* | *Isyna1* |
| *Fosb* | *Cryab* | *Exoc3l4* | *Nnt* | *Kif20a* | *Shmt1* |
| *Grem2* | *Cdk6* | *Hsd11b2* | *Hmmr* | *Dhcr24* | *Mylpf* |
| *Zdhhc14* | *Olr1* | *Pla2g4f* | *Cenpf* | *Nup210* | *Rbpms* |
| *Klf4* | *P2rx7* | *Isg20* | *Ccnb1* | *Klhdc8b* | *Igfbp2* |
| *Per2* | *Cobll1* | *Ern1* | *Dapk1* | *Tmem64* | *Sgms1* |
| *Ehbp1l1* | *Foxc1* | *Ins2* | *Rhebl1* | *Dnajc22* | *Rhpn2* |
| *Inpp4b* | *Rora* | *Nos2* | *Ptgfrn* | *Matn2* | *Cdca2* |
| *S100a14* | *Ddit3* | *Esam* | *Cdca8* | *Slc17a9* | *Nusap1* |
| *Zc3h12c* | *Ahr* | *Ell2* | *Mdk* | *Suclg2* | *Cdk1* |
| *Pag1* | *Bace2* | *Prdm1* | *Trim59* | *Nek2* | *Aurkb* |
| *Rnf222* | *Slc28a2* | *Pkp2* | *Bub1* | *Abhd14a* | *Acy1* |
| *Esrrg* | *Slc9a2* | *Pik3ap1* | *Bc055324* | *Cdc42ep2* | *Hoxc13* |
| *Ablim2* | *Il1rn* | *Tfpi* | *Cdc25b* | *Fbxo5* | *Laptm4b* |
| *Unc93a* | *Dhrs9* | *Cdkn1c* | *Bora* | *Ncapg2* | *Foxm1* |
| *Il1r2* | *Aqp3* | *Arhgef37* | *Ndc80* | *Cdc20* | *Pttg1* |
| *Gas7* | *Ano1* | *Bcar3* | *Cenpa* | *Tpx2* | *Dlgap5* |
| *Plin2* | *Pxdc1* | *Fads3* | *Vsig10* | *Cenpe* | *Nr1h3* |
| *Tram2* | *Lgals9* | *Siah2* | *Top2a* | *Slc25a13* | *Ralgps2* |
| *Rassf8* | *Slc7a2* | *Slc30a1* | *Slc25a23* | *Cdc42ep2* | *Mdk* |
| *Osmr* | *Fos* | *Adamts1* | *Rdm1* | *Upp1* | *Rhpn2* |
| *Lcor* | *Fam102b* | *Cyth1* | *Arhgap4* | *Mapt* | *Rhebl1* |
| *Arfgap3* | *Gdpd5* | *Gpr1* | *Igfbp2* | *Dapk1* | *Arhgef25* |
| *Bin2* | *App* | *Baz2b* | *Fdft1* | *Mex3a* | *Morc4* |
| *Tsc22d2* | *Optn* | *Fam214b* | *Rpp25* | *Ucp2* | *L1td1* |
| *Plac8* | *Rsad2* | *Tsc22d3* | *Vangl1* | *Trim59* | *Adamts7* |
| *Gpr137b* | *Slc7a11* | *Asprv1* | *Dok4* | *Ift80* | *Bc055324* |
| *Arhgap30* | *Plxdc2* | *Tlcd2* | *Hmgcs1* | *Hdac7* | *Slc1a5* |
| *Letm2* | *Plac1* | *Pdlim2* | *Zranb3* | *Epha1* | *Fgfr1* |
| *St3gal1* | *Hspb8* | *Pam* | *Hoxc13* | *Ptgfrn* | *Tmco6* |
| *Dnajb9* | *Mbnl3* | *E2f2* | *Ccna2* | *Dlgap5* | *Grip1* |
| *Optn* | *Snx9* | *Tns3* | *Acy1* | *Sox12* | *Slc25a18* |
| *Mt2* | *Ube2q2* | *Efhd1* | *Ndc80* | *Phc1* | *Parpbp* |
| *Zbtb38* | *Dock9* | *Frmd4a* | *Epb41l2* | *Sall4* |  |
| *Arfgap3* | *Rrbp1* | *Atl3* |  |  |  |
| *Mitf* | *Prkca* | *Rassf8* |  |  |  |
| *Fgd3* | *Tram2* | *Rnf223* |  |  |  |
| *Acot2* | *Ppp1r15a* | *Kctd11* |  |  |  |
| *Arl8a* | *Camk2n1* | *Bcar3* |  |  |  |
| *Mmp11* | *Sox13* | *Raph1* |  |  |  |
| *E2f8* | *Sat1* | *Tcp11l2* |  |  |  |
| *Mfsd2a* | *Mast4* | *Alas1* |  |  |  |
| *Pkib* | *Tgfbr2* | *Gpr160* |  |  |  |
| *Junb* | *Fam102b* | *Fhdc1* |  |  |  |
| *Golga2* | *Arhgef3* | *Neurl1b* |  |  |  |
| *Ubap1* | *Letm2* | *Inpp5d* |  |  |  |
| *Cyth1* | *Zfp488* | *Sp6* |  |  |  |
| *Synj1* | *Per1* | *Bc016579* |  |  |  |
| *Aloxe3* | *Metrnl* | *Phlda1* |  |  |  |
| *Rhobtb1* | *Gale* | *Secisbp2l* |  |  |  |
| *Gadd45g* | *Myc* | *Fam214b* |  |  |  |
| *Rdh13* |  |  |  |  |  |

**Table S4.** Common differentially expressed genes (upregulated and downregulated) identified in 4 days differentiated TGCs RNA-seq data and 3 days fused BeWo RNA-seq data.

| **Category** | **Name** | **Primers** |
| --- | --- | --- |
| Differential expression | *Pl1 F*  *Pl1 R*  *Tpbpa F*  *Tpbpa R*  *Fgfr2 F*  *Fgfr2 R*  *Err1 F*  *Err1 R*  *Psg18 F*  *Psg18 R*  *Itga2 F*  *Itga2 R*  *Psg29 F*  *Psg29 R*  *Ctsq F*  *Ctsq R*  *Gm11397 F*  *Gm11397 R*  *Sult1e1 F*  *Sult1e1 R*  *Cts7 F*  *Cts7 R*  *Pfpl F*  *Pfpl R*  *Duoxa1 F*  *Duoxa1 R*  *Foxh1 F*  *Foxh1 R*  *Drc7 F*  *Drc7 R*  *Palm3 F*  *Palm3 R*  *Gldc F*  *Gldc R*  *Tmem114 F*  *Tmem114 R*  *Sox3 F*  *Sox3 R*  *Capn6 F*  *Capn6 R*  *Aurka F*  *Aurka R*  *Aurkb F*  *Aurkb R*  *Ttk1 F*  *Ttk1 R*  *Incenp F*  *Incenp R*  *Cdca8 F*  *Cdca8 R*  *Actb F*  *Actb R* | 5'-GCAGATGTGTATAGGGAATT-3'  5'-GAAGTCCCTCCAGAATTAC-3'  5'-CTACAATCTTCCTAGTCATCC-3'  5'-CCTCTTCAAACATTGGGTGTA-3'  5'-TACGGGCCTGATGGGCTG-3'  5'-ACCACCATGCAGGCGATTAA-3'  5'-GCATCGAGCCTCTCTACATCA-3'  5'-GAGGCCGGACAGCTGTACT-3'  5'-ATCTGTGTGCGTCCTACTA-3'  5'-ATCAGCCCTTTGTACCAG-3'  5'-AGCTATCGAATTCGCAAG-3'  5'-TGCTATGCCGAACCTCAG-3'  5'-ACACCTGACATTGCAACC-3'  5'-CACACTTCGTAGGGGACAG-3'  5'-CCCAGTTTGGATGTCGAAT-3'  5'-CCCTAGGGAATTCTCCCTG-3'  5'-TCGCACTGAGTTTCATGTTTATTAT-3'  5'-TAAGTCAGCCTTGCTTCCATC-3'  5'-GCCAAAGATGTCGCCGTTTC-3'  5'-ACGGAACTTGCCCTTGCATA-3'  5'-GTGGTGCATGTTGGGCTTTT-3'  5'-CAGCCCTTGGTGCCATAAGA-3'  5'-GTGGAAACTGCTGCGGAAAG-3'  5'-AAGGGAGCGTTGACCAGTTT-3'  5'-CAGTGTGGACGTGGGACTGC-3'  5'-GCGCCATGCGAATGCCTCAT-3'  5'-AGAGATACCTACGGCATGAC-3'  5'-CTTATGGAAGCACCGATTAG-3'  5'-TGGCCATCGAGATCCCTACC-3'  5'-GCGGGAGAAGTTGTCTGCCA-3'  5'-AGCGCCCTCTATCGGCAG-3'  5'-GCCAACGTTCTCGGAGAGA-3'  5'-GGACATGGCCAACGCTTC-3'  5'-TTGGCTCGAGTCTGGACA-3'  5'-GAGAATGTGACGGTCAGCGA-3'  5'-GAAGCTCAAGAACCCGGTCA-3'  5'-GACTGGAAACTGCTGACCGAT-3'  5'-TGAGCAGCGTCTTGGTCTTG-3'  5'-CATTTTTGACACGCAGGCCA-3'  5'-TGGGGTCAGCATCGAGAGTA-3'  5'-CCTCGGCGTTCTCTGCTATGAGT-3'  5'-GCCTTTGGCTTGCGTTGTGTTT-3'  5'-ACCTTCGATGAGCAGCGGACTG-3'  5'-GCATGCACCGACCAGCCAAA-3'  5'-AGTGCCCAAGAGCCGTTCTCCA-3'  5'-AATCGCGTCTCGGTCTGGTCCT-3'  5'-AAGGTGTTGCAGGCCCGTGA-3'  5'-TTGATGCCGCCGCTCCTCTT-3'  5'-GCTGGAGCCGTCTCTGGTGAAA-3'  5'-TTGCTGTCTGCGAGAGGGCT-3'ss  5'-TCCGATGCCCTGAGGCTCTTT-3'  5'-TAGAAGCACTTGCGGTGCACGAT-3' |
| Alternative splice variants | *Tmem40 F*  *Tmem40 R*  *P2ry2 F*  *P2ry2 R*  *Ptprk F*  *Ptprk R*  *Tnnt1 F*  *Tnnt1 R*  *Garnl3 F*  *Garnl3 R*  [*Lrrfip1*](https://asia.ensembl.org/Mus_musculus/Gene/Summary?db=core;g=ENSMUSG00000026305;tl=RtX4JWuEZ2wfPrH3-5638840-748564076) *F*  [*Lrrfip1*](https://asia.ensembl.org/Mus_musculus/Gene/Summary?db=core;g=ENSMUSG00000026305;tl=RtX4JWuEZ2wfPrH3-5638840-748564076) *R*  *Lmo7 F*  *Lmo7 R*  *Dnmbp F*  *Dnmbp R*  *Kalrn F*  *Kalrn R*  *Asic1 F*  *Asic1 R* | 5'-ACAGTCAACACACAGCTCACC-3'  5'-TATATCGCTCCCGGGCCTT-3'  5'-TCCCTGGATGGACACGAAAAG-3'  5'-TTCAGGTCACTAACGGGTCC-3'  5'-CCGACTGCCGTGTTAGATGA-3'  5'-GTAAGTCGGCCACCCTGATG-3'  5'-AGGAGGAGCAGGCAGAA-3'  5'-CAAAGGAGGCACCACAGGA -3'  5'-AAATTTTTAAATCTCCTCGGTGACA-3'  5'-CATGACCTCATGCCCTTGGT-3'  5'-AATAGCCACCAACGGAGAGAC-3'  5'-TCCAAGATACCGTCCTCGGC-3'  5'-TGAACTCAAACTGCACGTCG-3'  5'-AGAGGCTCGAAAATCCGTTGT-3'  5'-GGGGCGATTCAGAGGGAAAT-3'  5'-GAGGATCAACAGAGGAGCCG-3'  5'-AGACAATGTGGATGGTGACCC-3'  5'-TGGATGTCTGAGTTGGCAGC-3'  5'-GCACCTGCAATGCTGTTACC-3'  5'-TGAACAATCCCATCTGGCCC-3' |
| Novel transcribed regions | *Asprv1 F*  *Asprv1 R*  *Zbtb7c F*  *Zbtb7c R*  *Sil1 F*  *Sil1 R*  *Ai50816 F*  *Ai50816 R*  *Specc1i F*  *Specc1i R*  *Map2k2 F*  *Map2k2 R*  *Fam179b F*  *Fam179b R*  *Eps8l1 F*  *Eps8l1 R*  *Snx29 F*  *Snx29 R*  *Gramd1c F*  *Gramd1c R* | 5'-GTCAGAATCTACATGAGGGGGT-3'  5'-CCCTTCTGCCAGAGAAATGCT-3'  5'-GGGAACTGTGTTCTGGGGATG-3'  5'-GGGGGCCAGAGCATCAATTC-3'  5'-CTGCTGAAGCTTTGCTCTTCC-3'  5'-CTCTAACCAGCGAGGGAGAC-3'  5'-CCTCGGAGGGAGTGGTAAAA-3'  5'-TTCCCAGACAGGTTGACCAAA-3'  5'-GTCCCTGCTATGGAAAGTGC-3'  5'-CCAAAGTGTTTCTGCCTCAGC-3'  5'-TGAGAATGGCCTTGCAAACAC-3'  5'-CCTATCATGGCCGATATGCCTC-3'  5'-ACAACACCCAGTGTAAGCAT-3'  5'-TTTTGCCTTGGGTGTTCCTT-3'  5'-GAACTATGGACCTCGCTGGG-3'  5'-GCTTGACAGATAGCTCGCTG-3'  5'-TGCAGTATGGGAAGCTCGTG-3'  5'-AGGGTCTCCTTCTTGGGGTT-3'  5'-AAGCAGAGCACCCTCCAAGT-3'  5'-ACTTCGTCGGACTTTCCCCA-3' |

**Table S5.** Primers used for experimental validation of differentially expressed genes, differentially expressed exons and novel identified exons.
